# Bithiazolidinylidene polymers: synthesis and electronic interactions with transition metal dichalcogenides†
†Electronic supplementary information (ESI) available. See DOI: 10.1039/c8sc01416g


**DOI:** 10.1039/c8sc01416g

**Published:** 2018-05-17

**Authors:** Ryan Selhorst, Peijian Wang, Michael Barnes, Todd Emrick

**Affiliations:** a Polymer Science and Engineering Department , 120 Governors Drive , Amherst , Massachusetts 01003 , USA . Email: tsemrick@mail.pse.umass.edu; b Department of Chemistry , University of Massachusetts Amherst , 710 North Pleasant Street , Amherst , MA 01003 , USA

## Abstract

We describe the synthesis of electron acceptors consisting of bithiazolidinylidene (BT) derivatives incorporated into solution processible polymers. Novel BT-containing polymers displayed p-doping behavior when in contact with the n-type transition metal dichalcogenide (TMDC) MoS_2_. A work function (WF) increase of MoS_2_, resulting from contact with BT polymers, was measured by Kelvin probe force microscopy (KPFM), representing the first example of polymer p-doping of MoS_2_, which is beneficial for advancing the design of electronically tailored TMDCs.

## Abstract 2

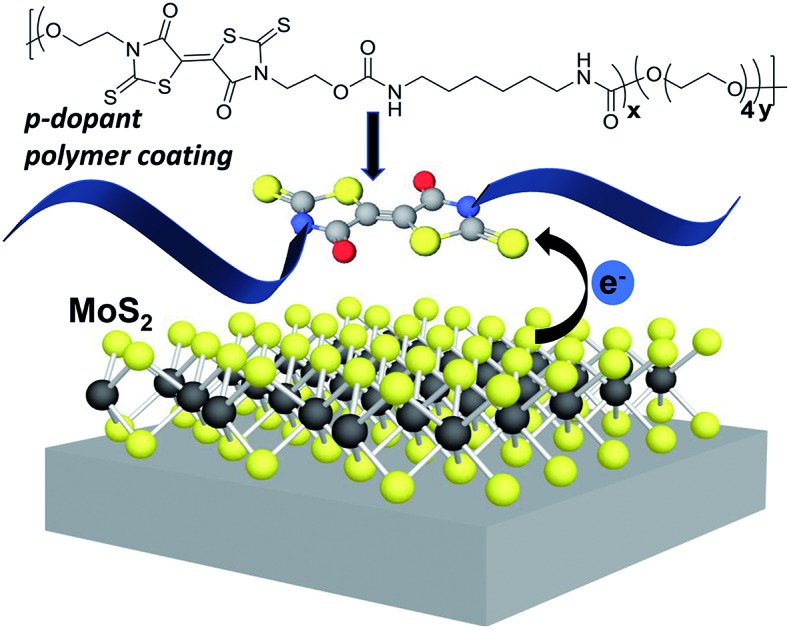
We describe the synthesis and characterization of polymers bearing sulfur-rich, electron-accepting bithiazolidinylidene (BT) groups, and probe their electronic impact on 2-D transition metal dichalcogenides (TMDCs).

## Introduction

2D transition metal dichalcogenides (TMDCs), such as molybdenum disulfide (MoS_2_) and tungsten diselenide (WSe_2_), are semiconductors with inherent bandgaps and the ability to transport both electrons and holes.^1^–^7^ Tuning TMDC electronic properties is desirable for their integration into 2D device architectures, such as p–n junctions^8^–^10^ and photovoltaics.^11^–^13^ Doping of TMDCs by the simple application of polymer coatings presents a solution processible method of tailoring macroscopic electronic properties. Current TMDC doping methods rely on embedding the TMDC lattice with atomic dopants, which requires high vacuum deposition. Implantation further induces defects in the form of sulfur vacancies on the basal plane, which irreversibly alters the 2D materials properties.^13^–^18^ Synthetic polymers, as soft materials dopants, are advantageous for their ability to electronically modify 2D semiconductors and open routes to patterning methodology. From prior investigation of MoS_2_ doping, electron donating polymers with a sulfur-rich structure promoted adhesion to the TMDC basal plane through sulfur–sulfur van der Waals interactions, and facilitated charge transfer at the TMDC/polymer interface.^19^

To date, reported examples of electron donating materials that decrease the work function of TMDCs (n-doping) are much more common than electron acceptors that increase the TMDC work function (p-doping). However, ready access to both p-and n-doping would afford fully tunable semiconductors and provide direct routes to p–n junctions containing both electron-rich and electron-deficient domains. To access the full range of TMDC electronics, we focus our efforts on p-doping by non-covalent physisorption without disturbing the inherent TMDC structure. For this study, we specifically prepared novel 5,5′-bithiazolidinylidene (BT) monomers to incorporate into polymers as p-dopants for MoS_2_.

**Scheme 1:**
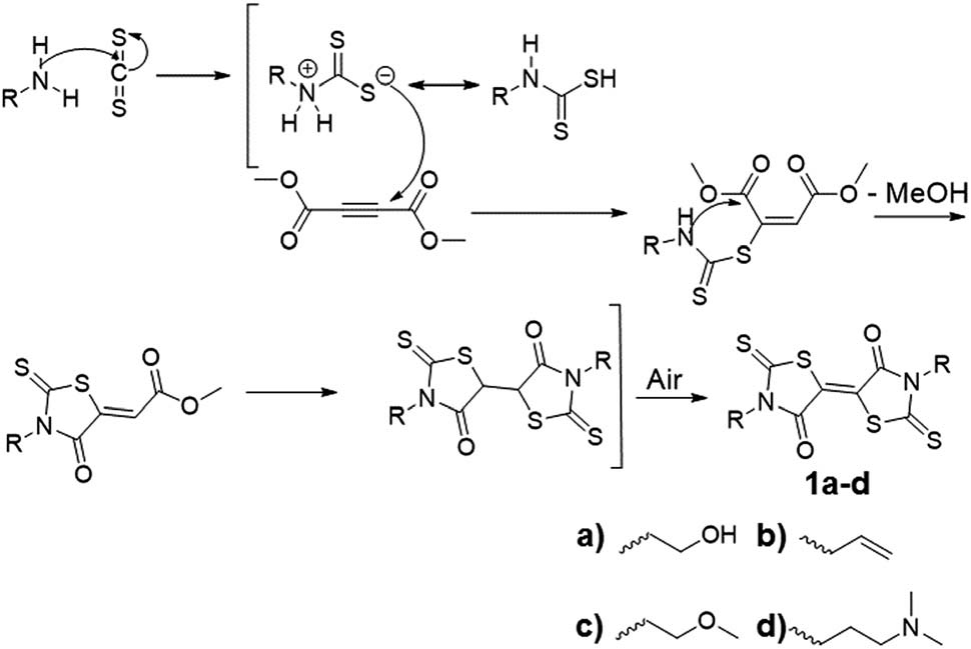
Synthesis of functional BT monomers.

BTs are sulfur-rich electron acceptors composed of fused rhodanine rings; they possess reversible redox potentials positioned at –0.20 V and –0.61 V, comparable to tetracyanoquinonedimethane (TCNQ).^20^ Due to the sulfur-rich structure, and its known electron accepting properties, BTs are anticipated to be effective p-dopants for sulfur-containing TMDCs. A one-step synthesis of BT-diones, reported by Nasiri, *et al.*, reacted aliphatic primary amines with carbon disulfide and dimethylacetylene dicarboxylate (Scheme 1).^21^ This method produced multi-gram quantities of BT derivatives in one step without the need for chromatographic purification. There are few known functional BT derivatives^22^,^23^ and no reports of BT-containing polymers. Thus, we sought to broaden the scope of BT chemistry by synthesizing BT derivatives capable of polymerization and subsequent solution processing as p-dopant coatings on TMDCs. We employed this one-step approach to yield difunctional BT monomers setup for integration into polymers by step-growth polymerization. Specifically, bishydroxyethyl BT **1a** was synthesized and incorporated into polyurethanes with the aim of p-doping MoS_2_ through its contact with thin film polymer coatings. The structure, stability, and energetics of the BT monomers and polymers were investigated spectroscopically and electrochemically and successful p-doping of monolayer CVD-grown MoS_2_ was confirmed by Kelvin probe force microscopy (KPFM), displaying an increase in work function after coating with these novel BT-containing polymer films.

## Results and discussion

### Polymer synthesis

**Scheme 2:**
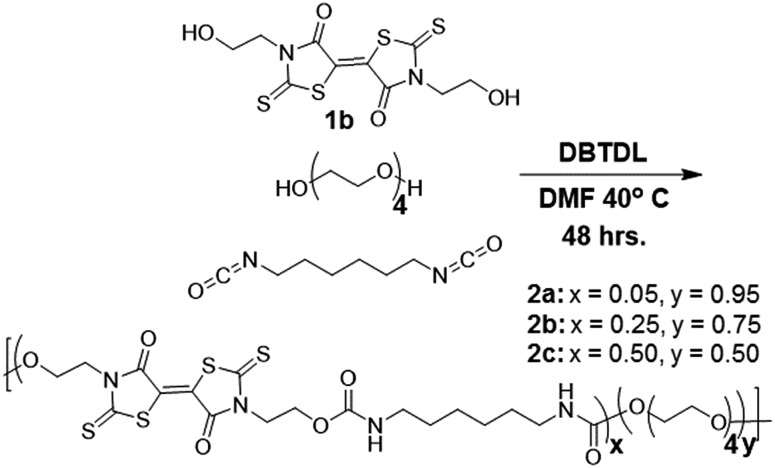
Synthesis of BT-containing polyurethanes.

**Fig. 1:**
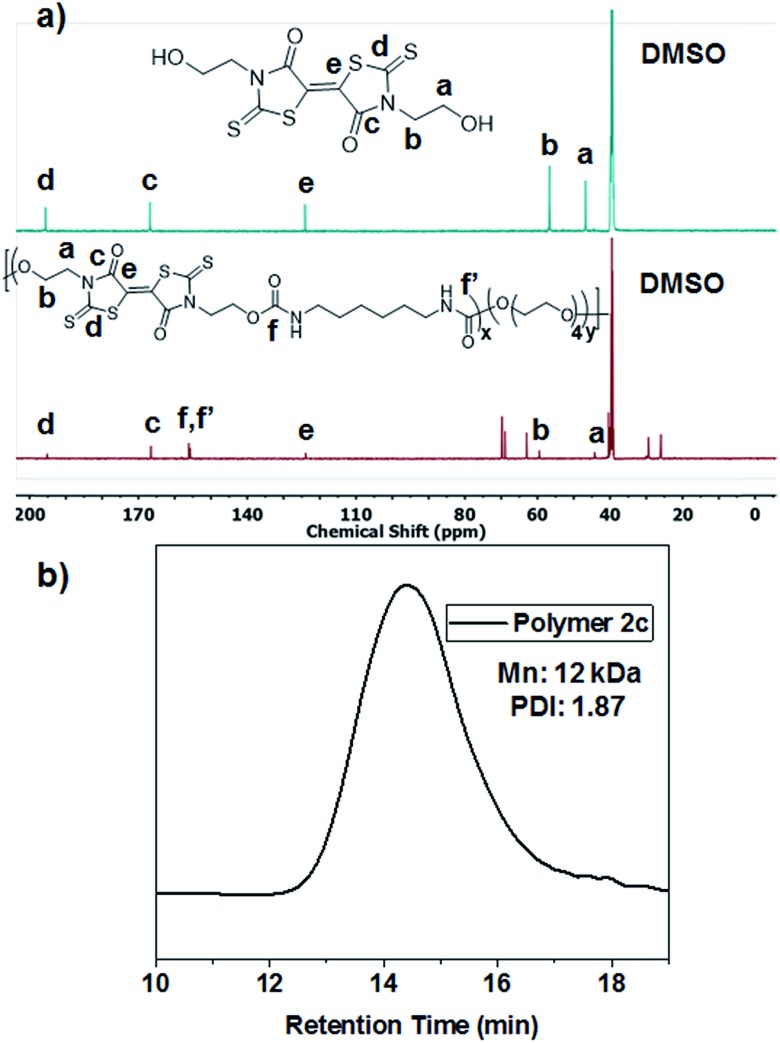
(a) ^13^C NMR spectra of BT-diol **1a** (top) and polymer **2c** (bottom) showing retention of the characteristic BT resonances after polymerization. (b) GPC chromatogram of polymer **2c**, showing monomodal molecular weight distribution.

Reacting the selected primary amines with carbon disulfide to form the corresponding dithiocarbamic acid, followed by slow addition of dimethylacetylene dicarboxylate at 0 °C, yielded functional BT monomers **1a–d** in yields approaching 50%. Attempts to use aniline as the primary amine were unsuccessful, likely due to its lower nucleophilicity in the cyclization step. The particularly facile accessibility of hydroxyethyl BT **1a** allowed its multi-gram scale synthesis and prompted our attempt to incorporate it into polyurethanes. Attempted homopolymerization of **1a** with hexamethylene diisocyanate (HMDI) in DMF led to insoluble product, with precipitation occurring before high conversion was achieved. Fortunately, copolymerization of **1a** with HMDI and tetraethyleneglycol performed at 40 °C, using dibutyltin dilaurate (DBTDL) as catalyst, produced soluble BT-polyurethanes in high yields (∼80–90%) (Scheme 2). Polymers **2a–c** were synthesized, with experimentally determined BT mole percentages corresponding closely to the monomer feed ratio. Polymer formation was monitored by ^1^H NMR spectroscopy, noting loss of the hydroxyl resonances at 4.9 ppm and appearance of urethane –NH signals at 7.0–7.1 ppm. The presence of BT groups in the polymers was further confirmed by ^13^C NMR spectroscopy, specifically noting the dithiocarbamate (195 ppm), BT carbamate (167 ppm), and BT alkene (124 ppm) resonances (Fig. 1a). Polymer molecular weight distributions, measured by gel permeation chromatography (GPC), were monomodal with molecular weights ranging from 12–30 kDa and polydispersity values of ∼2.0 (Fig. 1b), typical of step-growth polymerization.

### Spectroscopic characterization

**Fig. 2:**
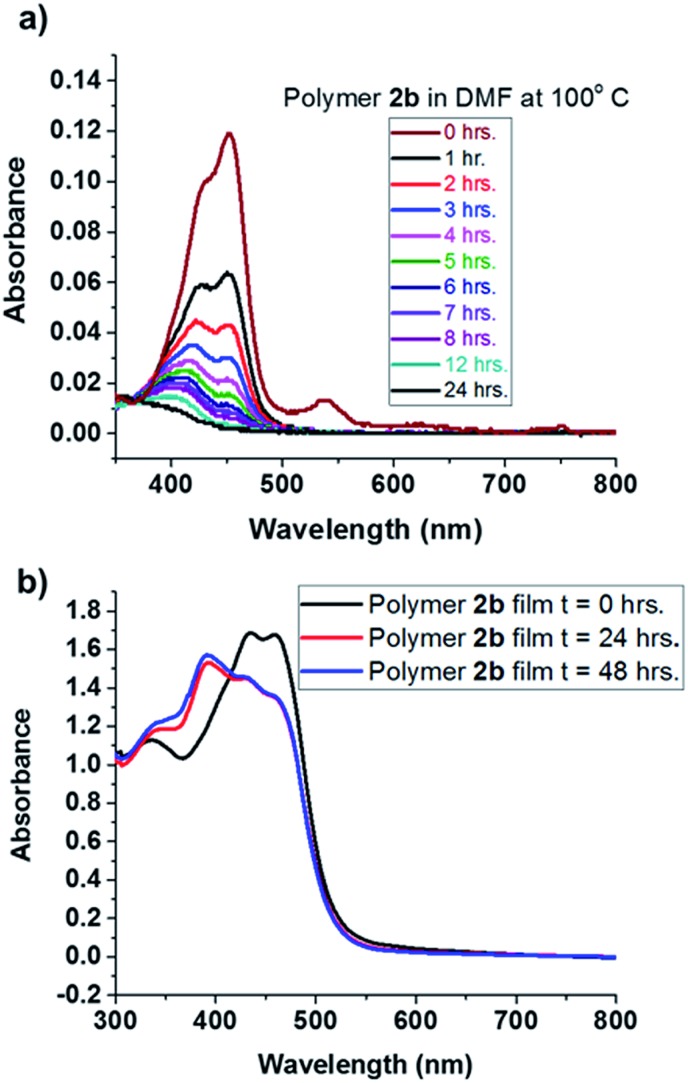
(a) UV-vis spectra of polymer **2b** heated at 100 °C in DMF for 24 hours; (b) thin film UV-vis spectra of polymer **2b**, on quartz, heated at 100 °C for 24 hours.

Interestingly, we observed that heating BT monomers as dilute solutions in DMF produced color changes, from orange to light yellow to colorless. The UV-vis spectrum of **1a** in DMF at room temperature showed absorption maxima at 440 and 425 nm for the 0–0 and 0–1 ground state transitions, respectively. Immediately upon heating at 100 °C in DMF, the absorbance intensity for BT at 440 nm decreased, and no new signals appeared (Fig. S22†). UV-vis spectroscopy of polymer **2b** in DMF showed similar quenching behavior to that of **1a**, with signatures fully diminishing after 24 hours (Fig. 2a). Notably, UV-vis spectra of thin films of polymer **2b** displayed no decrease in absorbance after 2 days at 100 °C on a quartz slide. A new peak appearing at 380 nm (Fig. 2b) is attributed to morphological changes in the thin film. Photoluminescence spectra of the MoS_2_ films before and after BT-doping (in particular the red-edge) shows spectral modification consistent with p-type doping; specifically the enhancement/suppression of PL emission to the red of the main PL peak associated with trion emission in MoS_2_.^24^ NMR spectroscopy of the thin film, after heating, confirmed the absence of chemical degradation, showing that the BT moiety is stable in the bulk, an important prerequisite to advancing its utility when embedded in thin polymer films.

Polyurethanes consisting of hard and soft segments undergo phase separation upon heating into domains rich in either hard or soft segments.^25^ Here, BT is the hard segment and tetraethylene glycol is the soft segment. To understand morphological changes in BT polymers that may alter spectroscopic signatures, small angle X-ray scattering (SAXS) was performed on a thin film of polymer **2b** before and after heating at 100 °C for 24 hours (ESI Fig. S26†). A broad peak, indicative of microphase separation in polyurethanes showed a domain size of 5 nm for the BT rich phase. After heating, the peak shifted to lower *q* values, resulting in a domain size of 10 nm for the BT-rich phase.

The differing stability observed in dilute solution and thin films led us to investigate solution stability of BT-based structures by NMR spectroscopy. A 0.01 M solution of monomer **1b** in DMF heated at 100 °C for two days yielded multiple degradation products. ^1^H NMR spectroscopy of the crude reaction mixture confirmed retention of the allylic protons, and showed new methylene resonances at 4.7 ppm. ^13^C NMR spectroscopy displayed the expected thiocarbonyl peak (195 ppm), and new carbonyl, allyl, and olefinic carbons suggesting a break in symmetry of the BT moiety (Fig. S18 and S19†). Further studies would be needed to identify the degradation products; however, the thin film thermal stability is encouraging for proceeding with these studies.

### Kelvin probe force microscopy (KPFM)

**Fig. 3:**
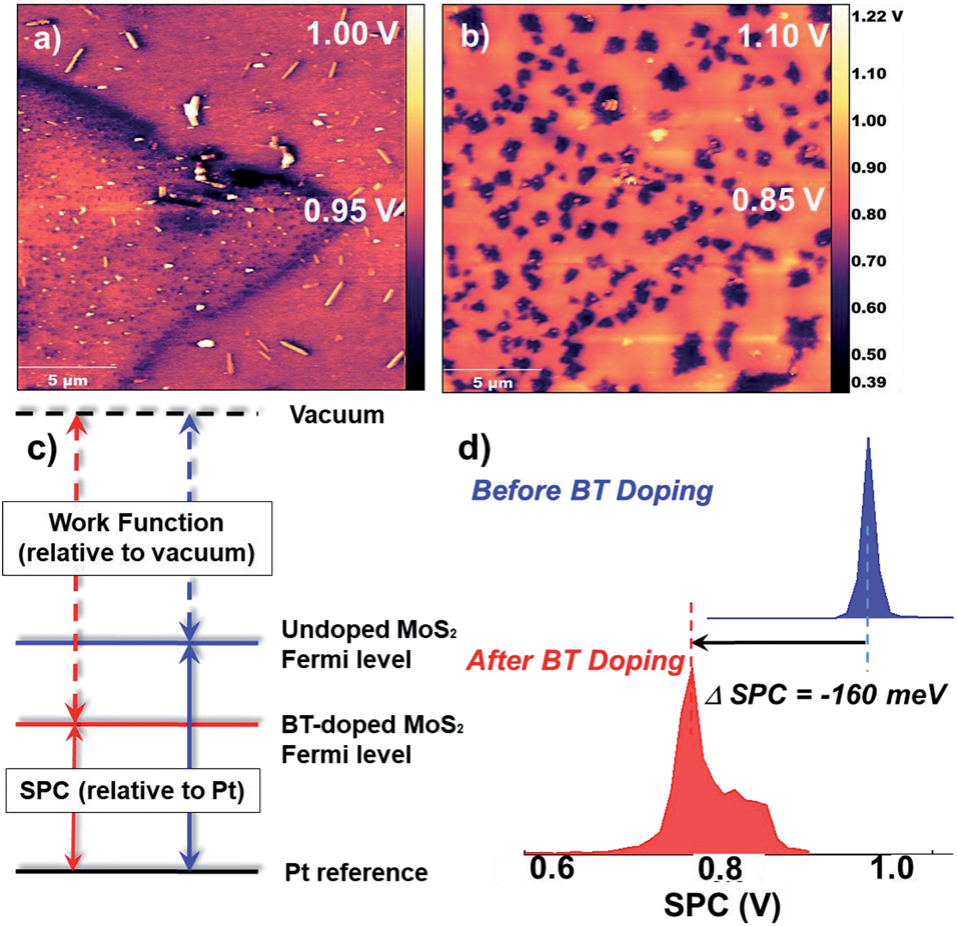
KPFM images of MoS_2_ on sapphire showing the surface potential contrast (SPC) (a) before coating with polymer **2b** and (b) after coating with polymer **2b**; (c) energy diagram depicting the work function increase after doping, relative to vacuum, manifest as a decrease in the SPC in the KPFM experiment; (d) plot of the shift in SPC before and after coating with polymer **2b**, displaying a 0.16 V decrease in SPC, indicating p-doping of MoS_2_.

To investigate the electronic influence of BT on TMDCs, KPFM was performed on a polymer-coated MoS_2_ monolayer. KPFM is a scanning probe technique that spatially measures the local electronic environment of the scanned surface. The output is an image showing the surface potential contrast (SPC) of the scanned area, which corresponds directly to a work function difference between the substrate and the conductive tip. Substrates of monolayer MoS_2_, grown by chemical vapor deposition on a sapphire (Al_2_O_3_) substrate, were initially scanned to analyze size and work function of the uncoated MoS_2_ flakes (see ESI† for height histograms). The substrate was composed mainly of monolayer and bilayer MoS_2_ with a roughly 1 nm step height difference corresponding to a single MoS_2_ layer (the height and SPC images also revealed the presence of impurities on the surface potentially dust; however, this did not alter the work function of MoS_2_). The SPC image of the uncoated substrate revealed work function values of 5.17 eV for monolayer flakes (Fig. 3a). Upon drop-casting a dilute solution (0.001 mg mL^–1^) of polymer **2b** onto the TMDC monolayer (resulting in roughly a 3 nm polymer coating) and rescanning the surface, a 0.16 V downshift in SPC was observed (Fig. 2b and c). In a control experiment, the work function of a thick (100 nm) polymer film measured as 5.5 eV, confirming that the measurements on the polymer-coated TMDCs reflect the impact of the polymer on the 2D material rather than simply the polymer itself (Fig. S29†). The *negative shift* in SPC observed for the thin polymer film on MoS_2_ correlates to a *work function increase* of MoS_2_, pushing the Fermi energy of MoS_2_ closer to the valance band edge, indicative of p-doping. After rinsing the substrate with chloroform, and scanning the same area, the work function reverted back to its initial value of 5.2 eV. To investigate the energetics and charge transfer of the BT/MoS_2_ system, were examined further with electrochemistry.

### Electrochemistry

**Fig. 4:**
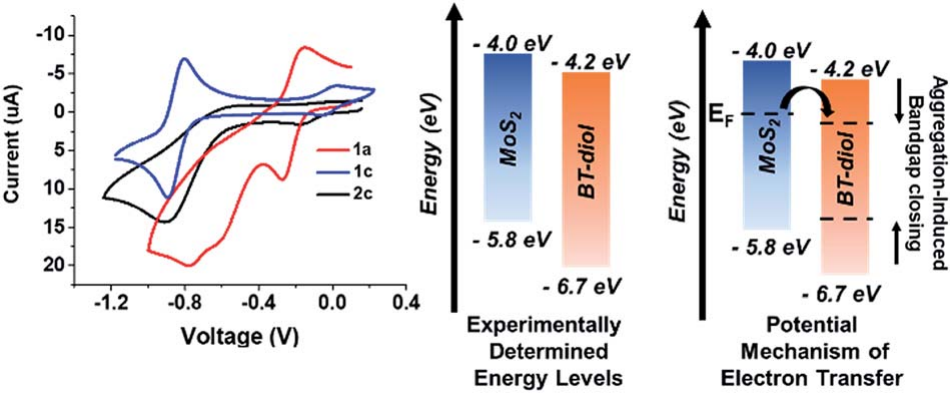
Left: cyclic voltammograms of BT-containing compounds **1a**, **1c**, and polymer **2c**. Center: energy band diagram of MoS_2_ and BT-diol (**1a**) with values estimated from CV and UV-vis. Right: potential mechanism for BT p-doping of MoS_2_ in which aggregation lowers the bandgap of BT allowing thermally excited electrons to transfer from MoS_2_ to BT.

Cyclic voltammetry (CV) was performed to examine the redox properties and energetics of the functional BT monomers and polymers. Fig. 4 (left) shows CV data acquired for compounds **1a**, **1c**, and polymer **2c**, in DMF using tetra-*n*-butylammonium hexafluorophosphonate as the electrolyte. The voltammogram of **1c** shows reversible redox potentials at –0.05 and –0.89 V, yielding a more negative reduction potential than electron acceptors such as TCNQ (*E*_1/2_^1^ = –0.06 V, CV shown in ESI†). However, BT-diol **1a** exhibited only one reversible reduction event, suggesting that the functional groups impede reduction to the dianion. Polymer **2c** (**2a** and **2b** shown in ESI†) showed a quasi-reversible first reduction and irreversible second reduction, similar to **1b**. From the onset of the reduction peaks observed by CV, and absorptions in the UV-vis spectra, the energy levels of the BT-containing structures were estimated. Fig. 4 (center) compares the experimentally determined energy levels of **1a** with that of monolayer MoS_2_. Interestingly, the MoS_2_/BT, donor/acceptor system is not ideal for ground state charge transfer of electrons from MoS_2_ to BT, a requirement for p-doping from a thin film. Many factors may contribute to a plausible doping mechanism including narrowing of the BT bandgap due to aggregation^26^ and/or an inherently n-doped MoS_2_ substrate, pushing the Fermi level closer to the conduction band of MoS_2_ (Fig. 4 right). This would provide a path for electron transfer to BT, increasing the work function of MoS_2_. However, further studies are required to identify the exact mechanism of charge transfer.

In summary, novel solution processible BT-containing polymers were synthesized, in which the BT-content was controlled by the selected monomer feed ratios. These step-growth polymerizations proceeded to high molecular weights, producing solution processible coatings for TMDCs. KPFM measurements of CVD-grown MoS_2_, after coating with BT-containing polymers, showed a work function increase of 0.16 eV over native MoS_2_, consistent with p-doping of the 2D material. This behavior is striking, as the experimentally determined energy levels of BT and MoS_2_ suggest unfavorable energetics for ground state electron transfer. However, the pronounced p-doping indicates a different doping mechanism than initially predicted such as aggregation-induced bandgap reduction and inherently doped substrate contributing to band structure changes in the BT/MoS_2_ system, warranting further investigation. While there are numerous examples of work function lowering (n-doping) materials for TMDCs, this work uncovers an unusual case of TMDC *p-doping*, pertinent for broadening the scope of 2D material devices. Moreover, these chalcogen-rich polymers can be used as a synthetic template for molecular design using other TMDCs to expand the scope of non-covalent doping routes.

## Associated content

Synthetic procedures, NMR, IR, and UV-vis spectra, cyclic voltammograms, X-ray spectra, thermal characterization, and GPC traces of all compounds can be found online in the ESI.†

## Author contributions

The manuscript was written through contributions of all authors. All authors have given approval to the final version of the manuscript.

## Conflicts of interest

There are no conflicts to declare.

## Supplementary Material

Supplementary information

## References

[cit1] Duan X., Wang C., Pan A., Yu R., Duan X. (2015). Chem. Soc. Rev..

[cit2] Manzeli S., Ovchinnikov D., Pasquier D., Yazyev O. V., Kis A. (2017). Nat. Rev. Mater..

[cit3] Splendiani A., Sun L., Zhang Y., Li T., Kim J., Chim C. Y., Galli G., Wang F. (2010). Nano Lett..

[cit4] Ma Y., Liu B., Zhang A., Chen L., Fathi M., Shen C., Abbas A., Ge Y., Mecklenburg M., Zhou C. (2015). ACS Nano.

[cit5] Choi J., Zhang H., Choi J. H. (2016). ACS Nano.

[cit6] Chow W. L., Yu P., Liu F., Hong J., Wang X., Zeng Q., Hsu C. H., Zhu C., Zhou J., Wang X., Xia J., Yan J., Chen Y., Wu D., Yu T., Shen Z., Lin H., Jin C., Tay B. K., Liu Z. (2017). Adv. Mater..

[cit7] Liu B., Abbas A., Zhou C. (2017). Adv. Electron. Mater..

[cit8] Cheng R., Li D., Zhou H., Wang C., Yin A., Jiang S., Liu Y., Chen Y., Huang Y., Duan X. (2014). Nano Lett..

[cit9] Li H. M., Lee D., Qu D., Liu X., Ryu J., Seabaugh A., Yoo W. J. (2015). Nat. Commun..

[cit10] Li M., Shi Y., Cheng C., Lu L., Liu Y., Tang H., Tsai M., Chu C., Wei K., He J., Chang W., Suenega K., Li L. (2015). Science.

[cit11] Shastry T. A., Balla I., Bergeron H., Amsterdam S. H., Marks T. J., Hersam M. C. (2016). ACS Nano.

[cit12] Sutar S., Agnihotri P., Comfort E., Taniguchi T., Watanabe K., Ung Lee J. (2014). Appl. Phys. Lett..

[cit13] Mahjouri-Samani M., Lin M. W., Wang K., Lupini A. R., Lee J., Basile L., Boulesbaa A., Rouleau C. M., Puretzky A. A., Ivanov I. N., Xiao K., Yoon M., Geohegan D. B. (2015). Nat. Commun..

[cit14] Kim E., Ko C., Kim K., Chen Y., Suh J., Ryu S. G., Wu K., Meng X., Suslu A., Tongay S., Wu J., Grigoropoulos C. P. (2016). Adv. Mater..

[cit15] Nipane A., Karmakar D., Kaushik N., Karande S., Lodha S. (2016). ACS Nano.

[cit16] Ren X., Ma Q., Fan H., Pang L., Zhang Y., Yao Y., Ren X., Liu S. F. (2015). Chem. Commun..

[cit17] Wang Y., Qi L., Shen L., Wu Y. (2016). J. Appl. Phys..

[cit18] Yang S., Tongay S., Yue Q., Li Y., Li B., Lu F. (2014). Sci. Rep..

[cit19] Selhorst R. C., Puodziukynaite E., Dewey J. A., Wang P., Barnes M. D., Ramasubramaniam A., Emrick T. (2016). Chem. Sci..

[cit20] Jaeger C., Bard A. (1979). J. Am. Chem. Soc..

[cit21] Nasiri F., Zolali A., Asadbegi S. (2016). J. Heterocycl. Chem..

[cit22] Filatre-Furcate A., Higashino T., Lorcy D., Mori T. (2015). J. Mater. Chem. C.

[cit23] Le Gal Y., Rajkumar M., Vacher A., Dorcet V., Roisnel T., Fourmigué M., Barrière F., Guizouarn T., Lorcy D. (2016). CrystEngComm.

[cit24] Mouri S., Miyauchi Y., Matsuda K. (2013). Nano Lett..

[cit25] Li Y., Gao T., Liu J., Linliu K., Desper C. R., Chu B. (1992). Macromolecules.

[cit26] Refaely-Abramson S., Sharifzadeh S., Jain M., Baer R., Neaton J. B., Kronik L. (2013). Phys. Rev. B: Condens. Matter Mater. Phys..

