# Supplementary material for: Bithiazolidinylidene polymers: synthesis and electronic interactions with transition metal dichalcogenides
Source: Chem Sci. 2018 May 17;9(22):5047–51. doi: 10.1039/c8sc01416g (PMC5994791; doi:10.1039/c8sc01416g)

Electronic Supplementary Material (ESI) for Chemical Science.
This journal is © The Royal Society of Chemistry 2018

Electronic Supplementary Information (ESI)

Bithiazolidinylidene Polymers: Synthesis and Electronic Interactions with Transition Metal Dichalcogenides

Ryan Selhorst[1], Peijian Wang[2], Michael Barnes[2], and Todd Emrick[1]*

[1]Polymer Science and Engineering Department, 120 Governors Drive, Amherst, Massachusetts 01003, United States. [2]Department of Chemistry, University of Massachusetts, Amherst, 710 North Pleasant Street, Amherst, MA 01003, United States. Email: tsemrick@mail.pse.umass.edu.

Correspondence to: tsemrick@mail.pse.umass.edu

- *Materials & Instrumentation* **S2**
- *Synthetic Procedures & Materials Characterization* **S2**
- *Infrared Spectroscopy* **S11**
- *Gel Permeation Chromatography* **S12**
- *NMR Degradation Study* **S13**
- *Electrochemistry* **S14**
- *Thermal Characterization* **S17**
- *UV-Vis* **S18**
- *X-ray Scattering* **S19**
- *Kelvin Probe Force Microscopy* **S20**

Experimental:

Materials. Dimethylacetylene dicarboxylate (96%) was purchased from TCI chemicals. Ethanolamine (95%), allyl amine (98%), 2-methoxyethylamine (95%), tetraethyleneglycol (99%), hexamethylene diisocyanate (HMDI) (98%), carbon disulfide (98%), dibutyltin dilaurate (DBTDL) (95%), chloroform (99%), dimethylformamide (DMF) (99.8%), dimethylsulfoxide (DMSO) (99.98%), and tetra *n*-butylammonium hexafluorophosphonate ($TBAPF_6$) (98%) were purchased from Sigma Aldrich. All chemicals were used as received without any further purification. MoS_2, CVD grown on Si/SiO_2 and sapphire (Al_2O_3) substrates, was purchased from SixCarbon Technologies and used as received.

Instrumentation. ^1H NMR (500 MHz) and ^{13}C NMR (125 MHz), spectra were obtained using a Bruker Ascend^{TM} 500 spectrometer equipped with a Prodigy cryoprobe. Gel permeation chromatography (GPC) was carried out in DMF with 0.01 M LiCl at 50 °C against PEO calibration standards. Samples were run using a flow rate of 1.0 mL/min with a Sonntek K-501 pump, one 50 × 7.5 mm PL gel mixed guard column, one 300 × 7.5 mm PL gel 5 μm mixed C column, one 300 × 7.5 mm PL gel 5 μm mixed D column, and using a Knauer refractive index detector (K-2301) and an Alltech model 3000 solvent recycler. UV-Vis measurements were carried out on a Shimadzu UV-2600 spectrophotometer equipped with a temperature controlled cell. All spectra were recorded using quartz cuvettes with a 1 cm path length. Electrospray ionization (ESI) mass spectrometry data were obtained using a Bruker MicroTOF II mass spectrometer. ESI-MS employed chloroform solutions of 1 mg/mL. Cyclic voltammetry was carried out using an Epsilon Basic electrochemical workstation with C3-cell stand (BASi Instruments). Thermogravimetric analysis (TGA) was performed on a Q500 (TA instruments) thermogravimetric analyzer under nitrogen atmosphere. The temperature was swept from 30 ^oC to 800 ^oC at a temperature ramp of 10 ^oC/min. Fourier-transform infrared spectroscopy (FT-IR) spectra were recorded using a PerkinElmer Spectrum 100 equipped with an attenuated total reflectance (ATR) accessory. Domain spacings of BT polymer films were characterized using small and wide angle X-ray scattering (SAXS/WAXS) using a Ganesha SAXS/WAXS-LAB instrument with Cu Kα 0.154 nm line on SAXS or WAXS mode.

General procedure for the synthesis of ((*E*)-3,3'-substituted-5,5′-bithiazolidinylidene-4,4′-dione) To a 20mL scintillation vial, chilled to 0 ^oC in an ice bath, was added amine (2 eq.) in DMF. Carbon disulfide (2 eq.) was added dropwise (very exothermic) and the now yellow/orange solution was stirred for 10 minutes. Dimethylacetylene dicarboxylate (1 eq.) was then added dropwise, the now dark red/black solution was stirred for an additional 10 minutes. The solution was precipitated in an excess of methanol:water (1:1) to yield an orange crystalline precipitate. The orange solid was collected by vacuum filteration and dried under vacuum to give the product. **1a.** Yield: 40% ^1H NMR (500 MHz, DMSO) δ: 4.92 (t, J = 6.1 Hz, 1H), 4.12 (s, 2H), 3.66 (q, J = 6.0 Hz, 2H). ^{13}C NMR (126 MHz, DMSO) δ: 195.57, 166.77, 124.02, 56.66, 46.73. ESI-MS: calculated for $C_{10}H_{10}N_2O_2S_4$ [M+]: 348.9451, found: 348.9420. FT-IR ν (cm^{-1}) 3100-3500 (b, -OH), 2966-2822 (w, alkyl C-H), 1687,1703 (s, C=O), 1254 (m, C=S), 1067 (m, -N-C=S)

1b. Yield: 46%, ^1H NMR (400 MHz, DMSO) δ: 5.83 (ddt, J = 17.0, 10.5, 5.2 Hz, 1H), 5.29 – 5.05 (m, 2H), 4.64 (d, J = 5.2 Hz, 2H). ^{13}C NMR (101 MHz, DMSO) δ: 195.39, 166.74, 130.32, 124.75, 118.71, 46.64. ESI-MS: calculated for $C_{12}H_{10}N_2O_2S_4$ [M+]: 341.9631, found: 341.9681. FT-IR ν

(cm^{-1}) 2989-2990 (w, alkyl C-H), 3090-3000 (m, allyl C-H), 1695,1675 (s, C=O), 1640 (w, C=C), 1289 (m, C=S), 1025 (m, N-C=S).

1c. Yield: 50%, ^{1}H NMR (500 MHz, $CDCl_3$) δ: 4.35 (t, *J* = 5.6 Hz, 2H), 3.69 (t, *J* = 5.6 Hz, 2H), 3.33 (s, 3H). ^{13}C NMR (126 MHz, $CDCl_3$) δ 194.87, 167.10, 124.86, 68.22, 59.07, 43.79. ESI-MS: calculated for $C_{12}H_{14}N_2O_4S_4$ [M+]: 377.98, found [M+Na]: 400.973. FT-IR ν (cm^{-1}) 2995-2784 (w, alkyl C-H), 1687 (s, C=O), 1271 (m , C=S), 1060 (m, N-C=S)

1d. Yield: 35%, ^{1}H NMR (500 MHz, $CDCl_3$) δ: 4.20 (t, J = 7 Hz, 2H), 2.37 (t, J = 7 Hz, 2H), 2.19 (s, 6H), 1.87 (p, J = 7 Hz, 2H). ^{13}C NMR (126 MHz, $CDCl_3$) δ: 194.69, 167.10, 124.76, 56.98, 45.50, 43.43, 24.71. ESI-MS: calculated for $C_{16}H_{24}N_4O_2S_4$ [M+]: 432.08, found [M+H]: 433.085. FT-IR ν (cm^{-1}) 2985-2677 (m, alkyl C-H), 1691 (s, C=O), 1253 (m, C=S), 1073 (m, N-C=S).

General procedure for the synthesis of polyurethanes (2a-c)
To a flame-dried 25 mL round-bottomed flask went BT-diol and tetraethyleneglycol in DMSO. DBDTL and HMDI were added quickly and the mixture stirred at 40 °C for 48 hours. The viscous polymer solution was precipitated into methanol and collected by filtration to yield orange fibers. Further purification by dialysis was performed in THF using 3500 Da molecular weight cutoff dialysis membranes. The THF solutions were again precipitated in methanol, collected by vacuum filtration and dried overnight under vacuum.

2a. Yield: 90% ^{1}H NMR (500 MHz, DMSO) δ (ppm) 7.15-7.17 (m), 4.25 (s), 4.02-4.04 (t), 3.54-3.56 (t), 3.51 (s), 2.94 (q), 1.3-1.4 (m), 1.15-1.25 (m). ^{13}C NMR (126 MHz, DMSO) δ (ppm) 195.20, 166.59, 156.15, 156.09, 123.88, 69.79, 69.72, 68.92, 63.18, 60.23, 44.22, 40.74, 29.38, 25.97. FT-IR ν (cm^{-1}) 3321 (s, urethane N-H), 2988-2788 (s, alkyl C-H), 1682 (s, urethane C=O), 1250 (m, C=S), 1053 (m, N-C=S).

2b. Yield: 86%, ^{1}H NMR (500 MHz, DMSO) δ (ppm) 7.15-7.17 (m), 7.08 (t), 4.20-4.30 (s), 4.02-4.05 (t), 3.53-3.57 (t), 3.51 (s), 2.94 (q), 2.88 (q), 1.3-1.4 (m), 1.16-1.25 (m). ^{13}C NMR (126 MHz, DMSO) δ (ppm) 195.59, 167.03, 156.59, 156.23, 124.33, 70.25, 70.18, 69.38, 63.44, 59.93, 44.66, 40.62, 29.83, 26.42. FT-IR ν (cm^{-1}) 3320 (m, urethane N-H), 2988-2796 (m, alkyl C-H), 1685 (s, urethane C=O), 1253 (m, C=S), 1054 (m, -N-C=S).

2c. Yield: 84%, ^{1}H NMR (500 MHz, DMSO) δ (ppm) 7.15-7.20 (m), 7.08 (m), 4.20-4.30 (s), 4.02-4.10 (m), 3.53-3.57 (m), 3.48 (s), 2.90-3.00 (m), 2.80-2.90 (m), 1.3-1.4 (m), 1.16-1.25 (m). ^{13}C NMR (126 MHz, DMSO) δ (ppm) 195.59, 173.80, 167.03, 156.59, 156.22, 124.34, 70.25, 70.18, 69.38, 63.44, 59.93, 44.58, 40.64, 29.83, 26.43. FT-IR ν (cm^{-1}) 3331 (m, broad, urethane N-H), 2983-2814 (m, alkyl C-H), 1690 (s, urethane C=O), 1250 (m, C=S), 1052 (m, N-C=S).

Figure **S1**: ^{1}H NMR of compound 1a
* Represents residual DMSO
** Represents residual water in the solvent.

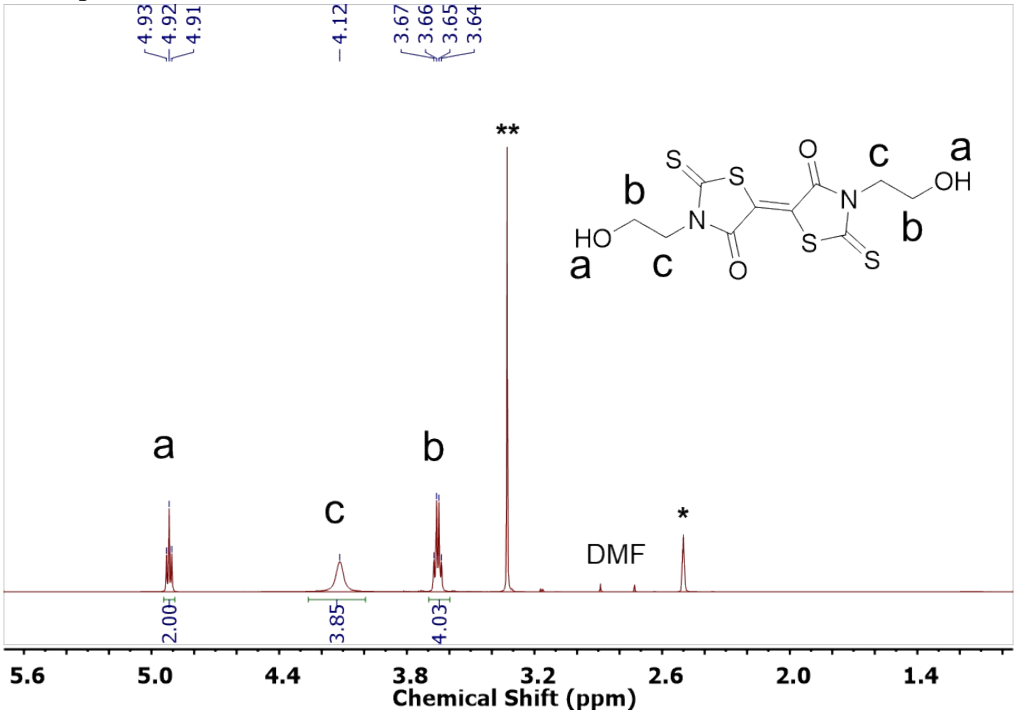

Figure **S2**: ^{13}C NMR of compound 1a
* Represents DMSO-d_6

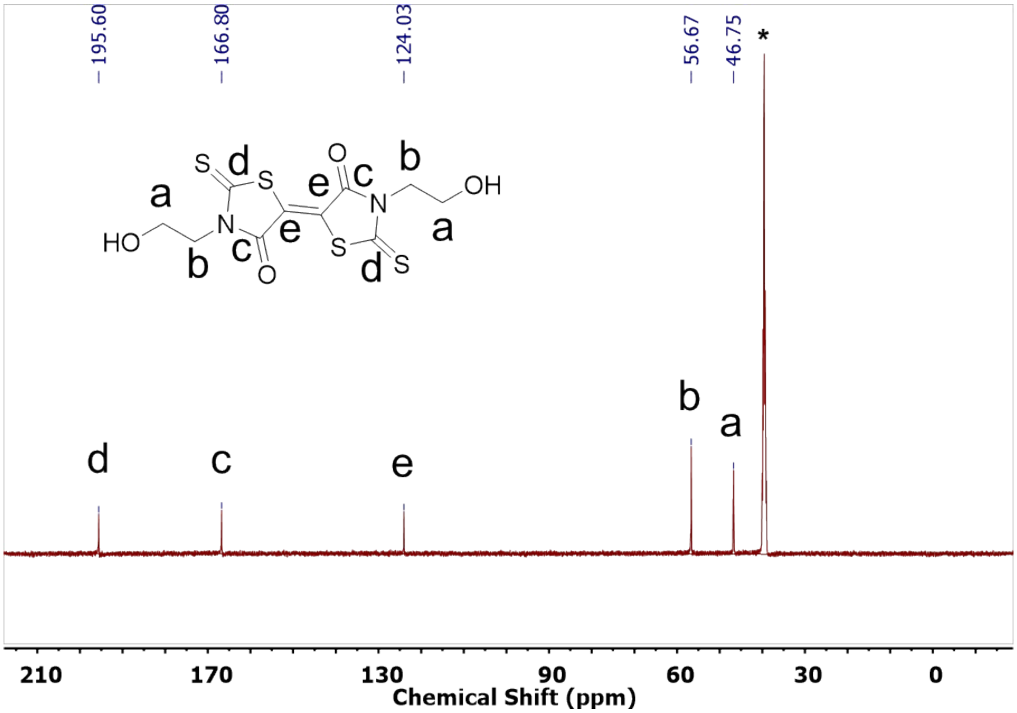

Figure **S3**: ^{1}H NMR of compound 1b
* Represents residual DMSO
** Represents residual water in the solvent

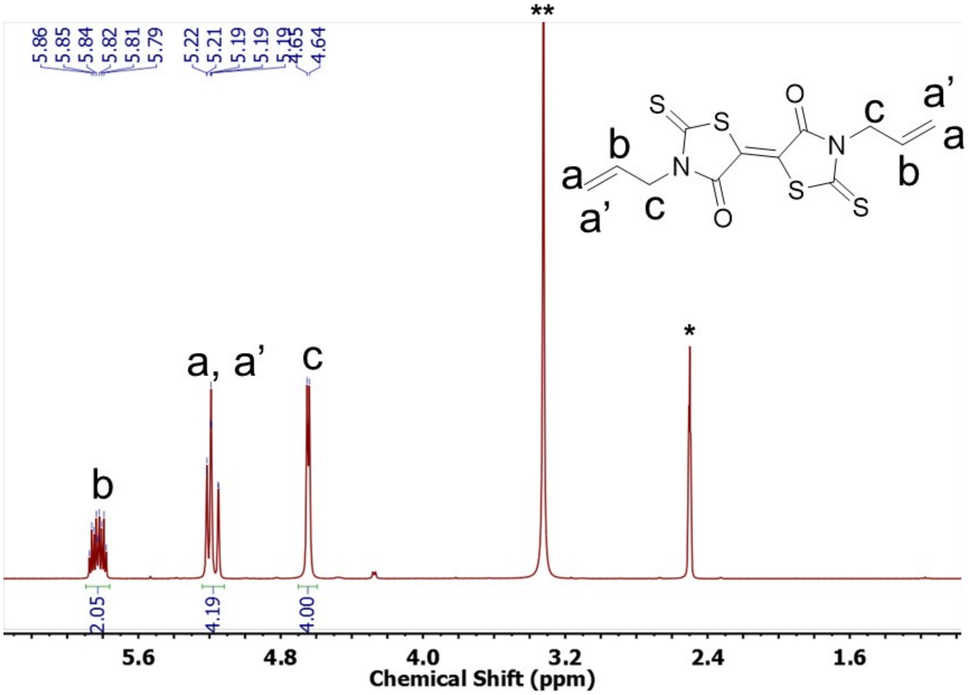

Figure **S4**: ^{13}C NMR of compound 1b
* Represents DMSO-d_6

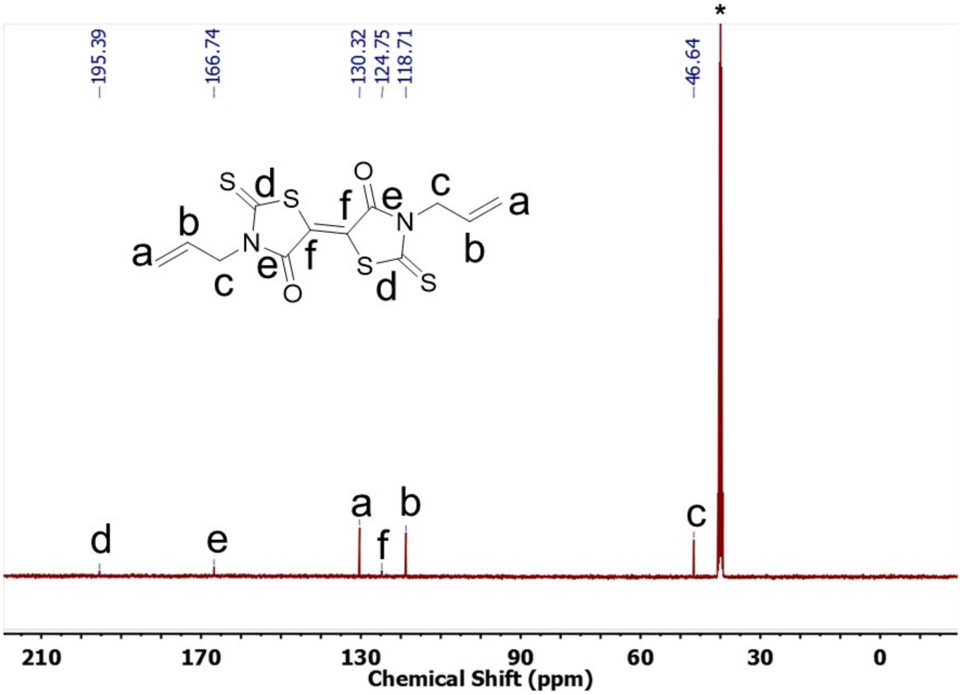

Figure **S5**: ¹H NMR of compound 1c
* Residual Chloroform
** Residual water in solvent
*** Tetramethylsilane

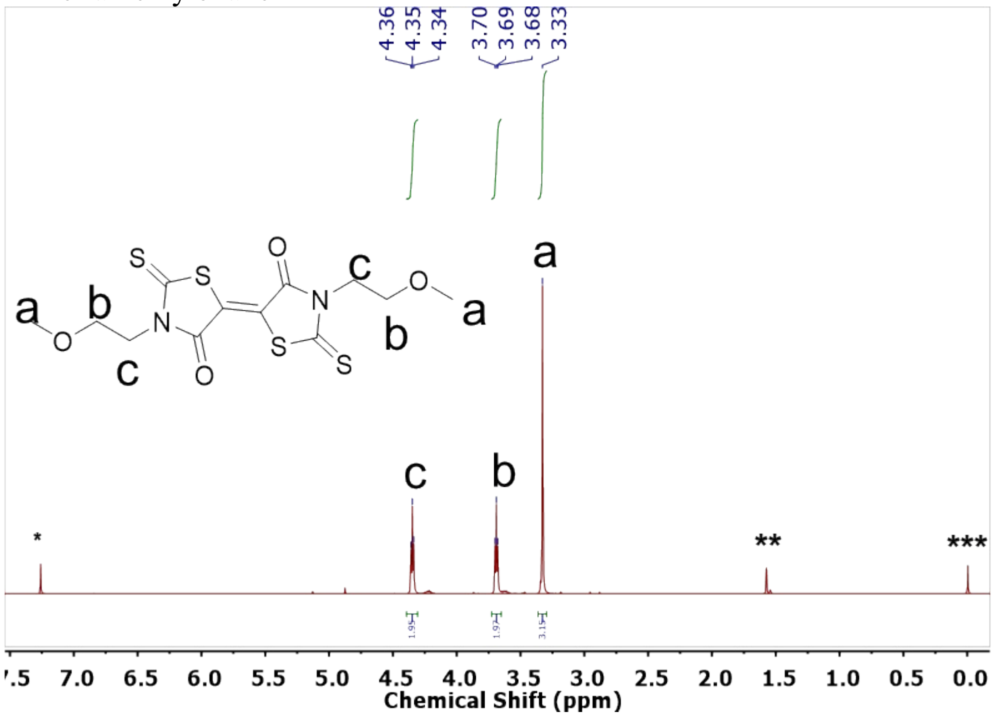

Figure **S6**: ¹³C NMR of compound 1c
*Residual Chloroform

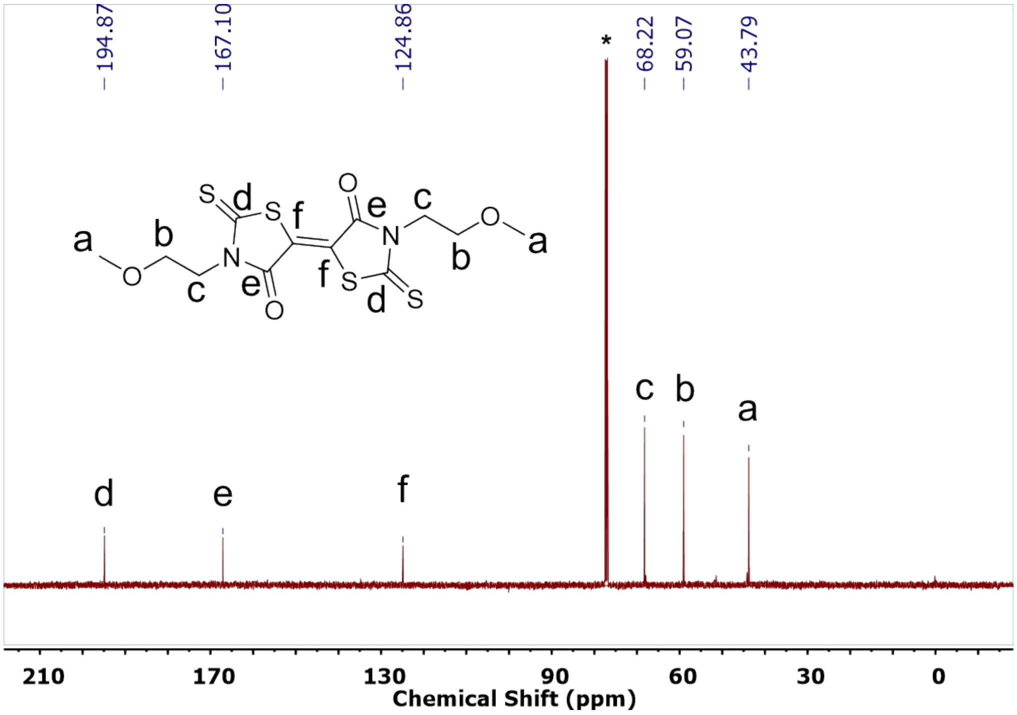

Figure **S7**: ^{1}H NMR of compound 1d
*Residual Chloroform

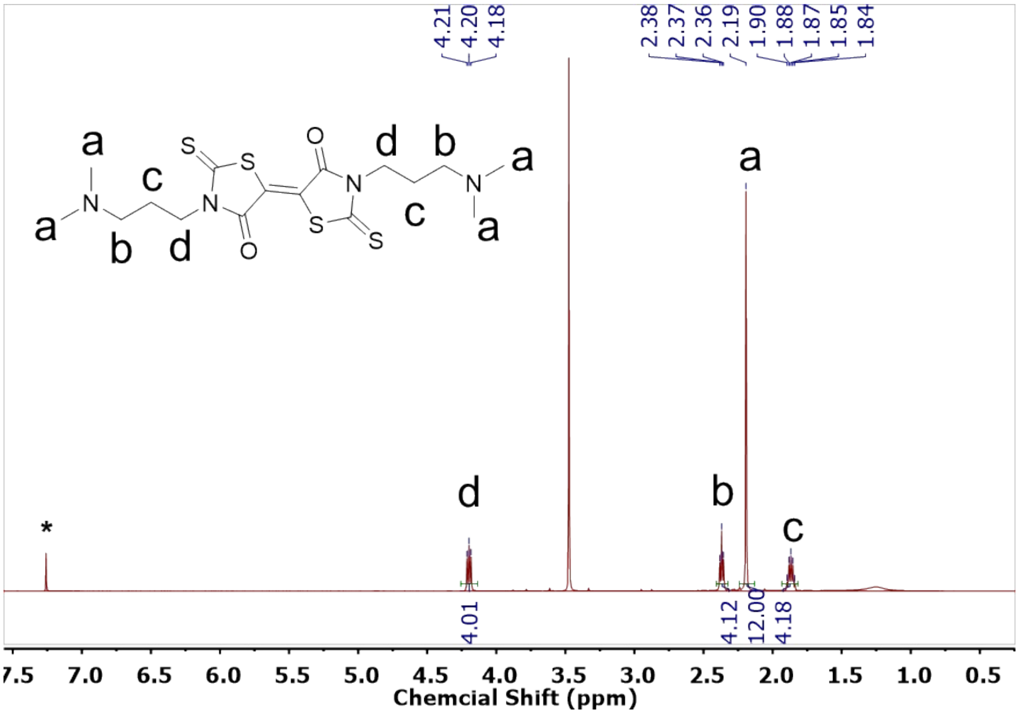

Figure **S8**: ^{13}C NMR of compound 1d
*Residual Chloroform

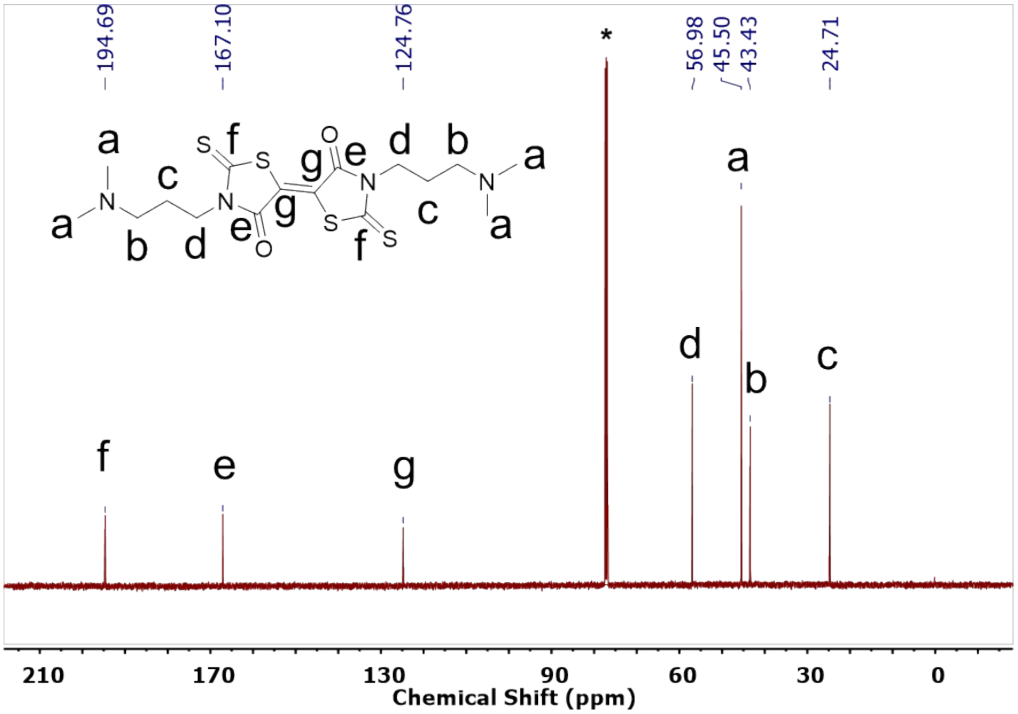

Figure **S9**: ^{1}H NMR of polymer 2a
* Residual DMSO
** Residual water in solvent
*** Tetraethyleneglycol peaks in polymer

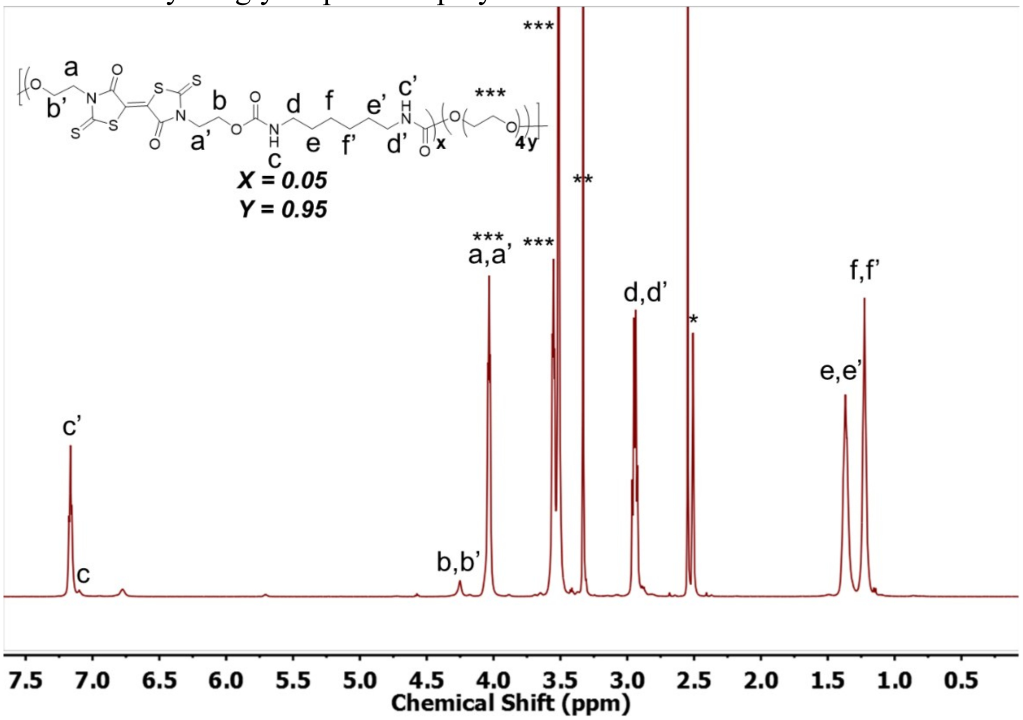

Figure **S10**: ^{13}C NMR of polymer 2a
* Residual DMSO
** Tetramethylsilane
*** Tetraethyleneglycol peaks in polymer

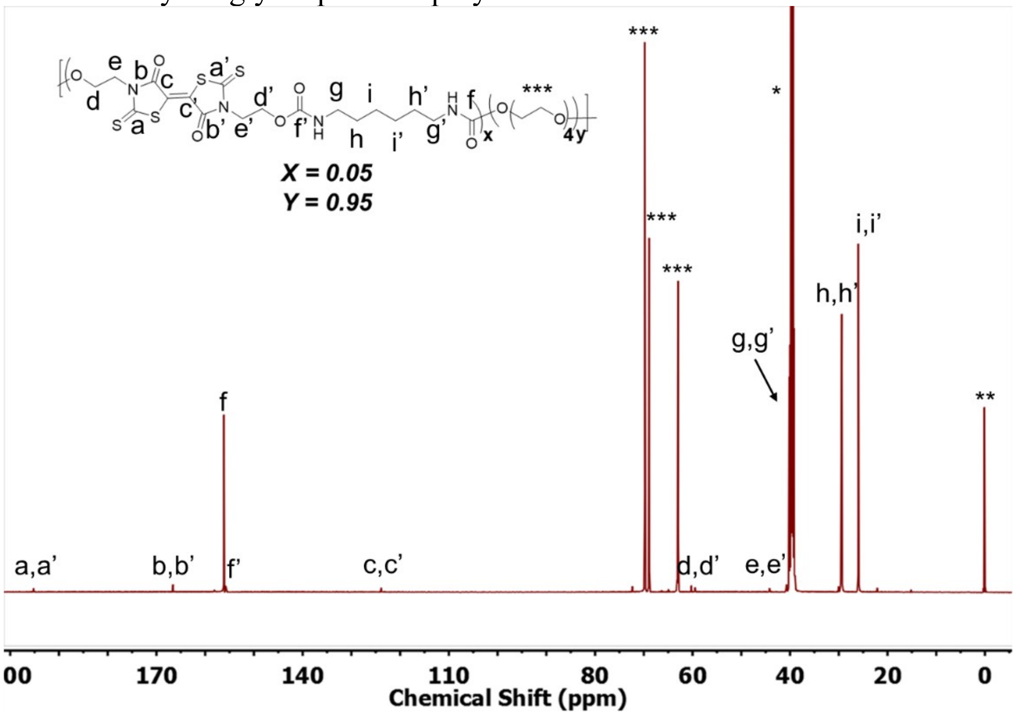

Figure **S11**: ^{1}H NMR of polymer 2b
* Residual DMSO
** Residual water in solvent
*** Tetraethyleneglycol peaks in polymer

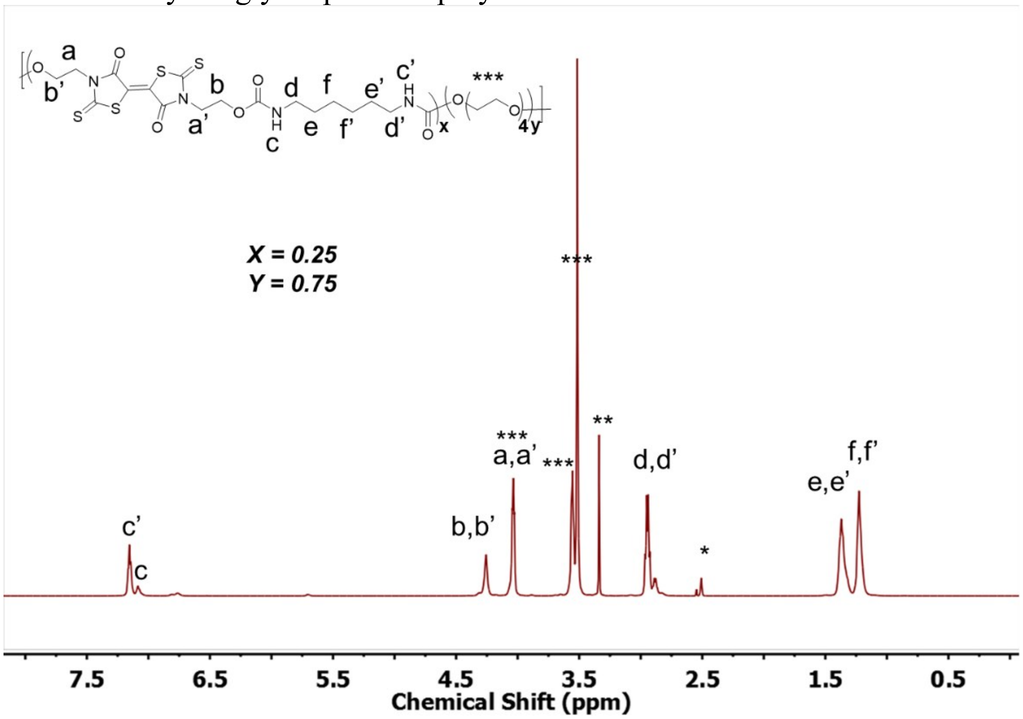

Figure **S12**: ^{13}C NMR of polymer 2b
* Residual DMSO
**Tetraethyleneglycol peaks in polymer

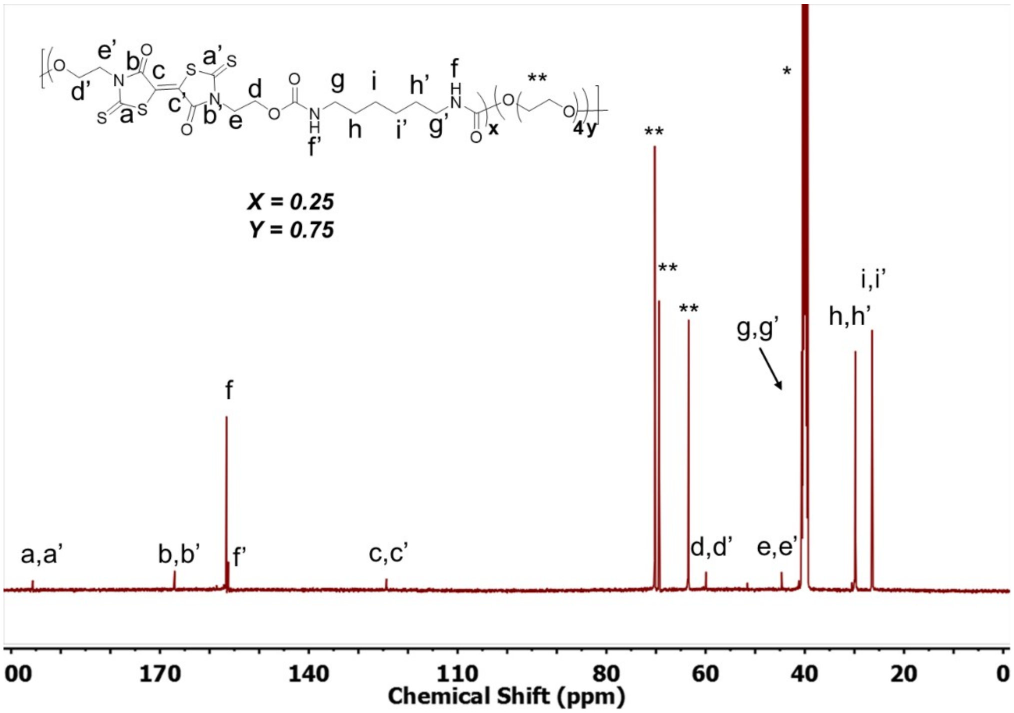

Figure **S13**: ^{1}H NMR of polymer 2c
* Residual DMSO
** Residual water in solvent
*** Tetraethyleneglycol peaks in polymer

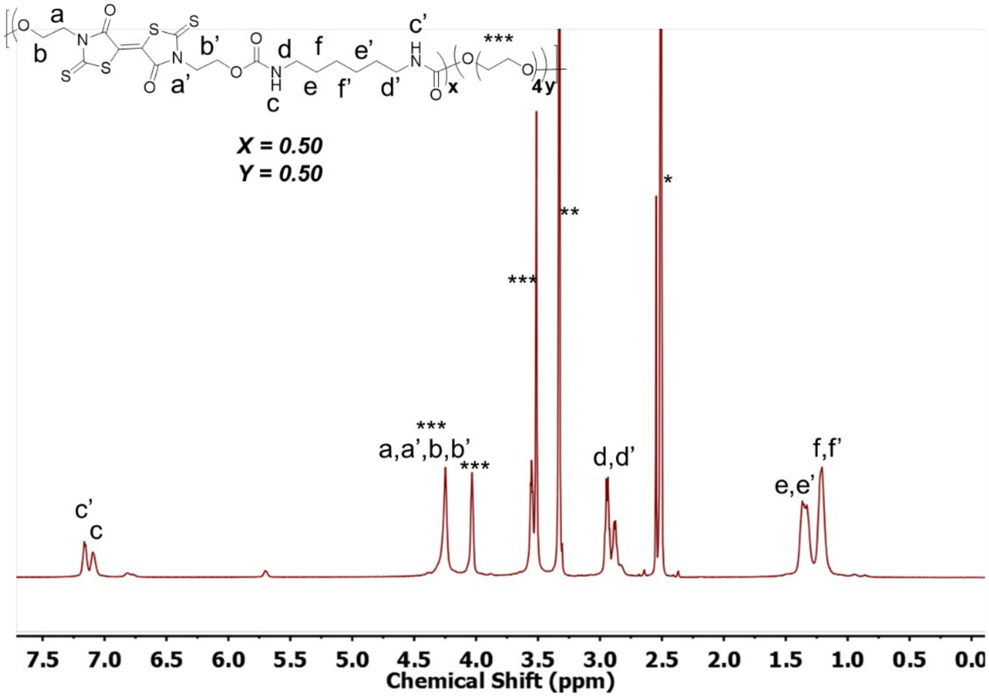

Figure **S14**: ^{13}C NMR of polymer 2c
* Residual DMSO
**Tetraethyleneglycol peaks in polymer

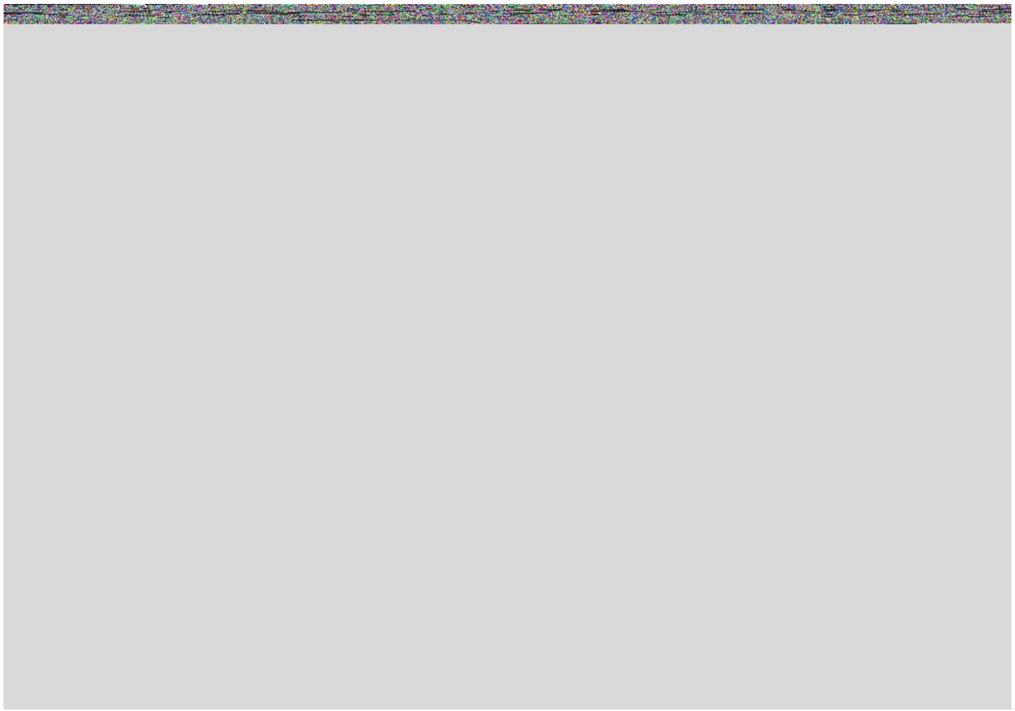

Infrared Spectroscopy (Figures S15)

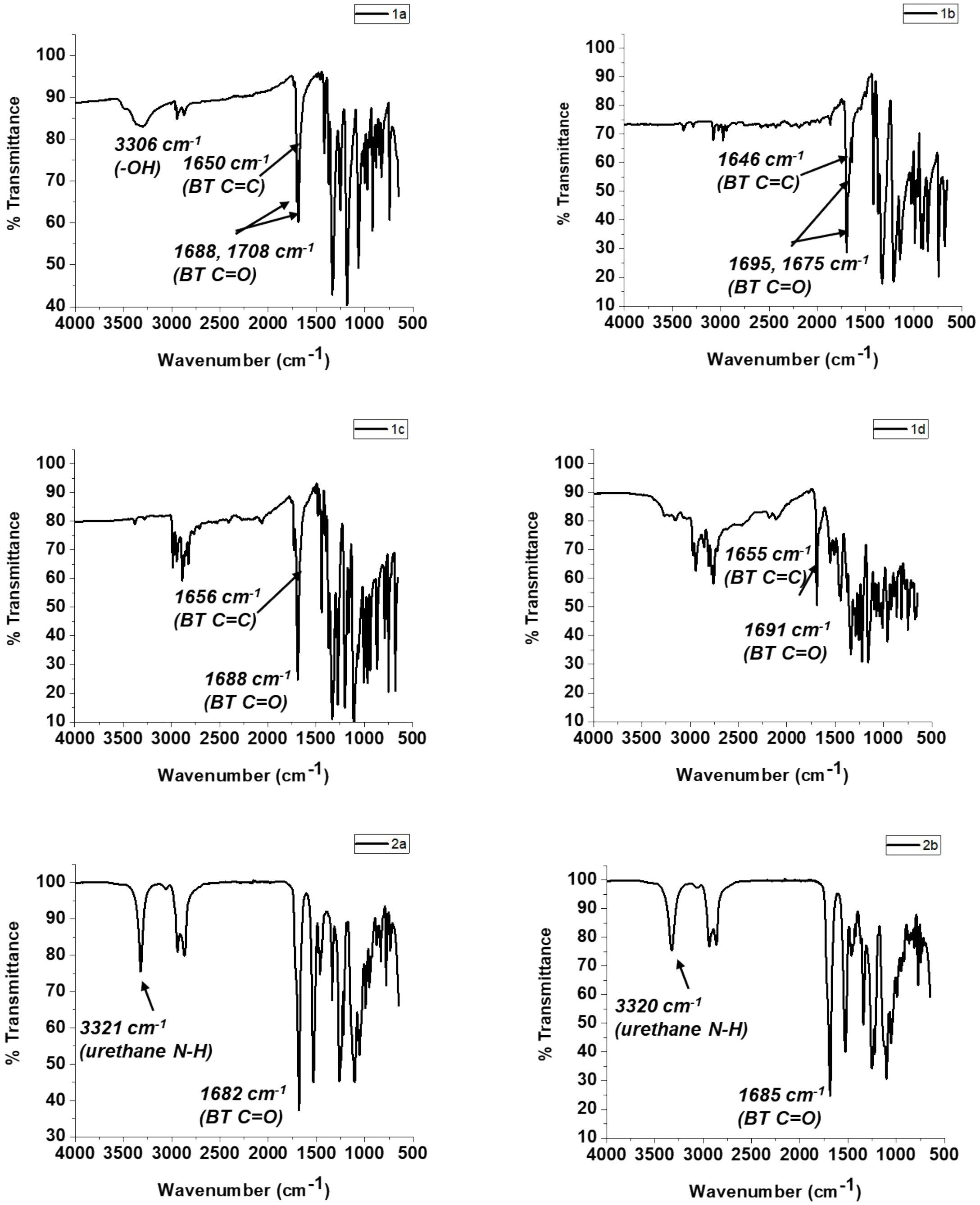

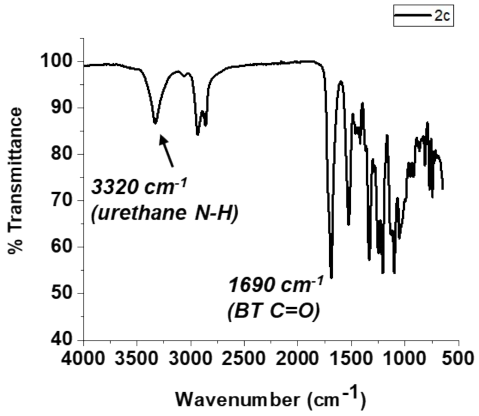

Gel Permeation Chromatography

Figure **S16**: GPC chromatograph of polymer 2a

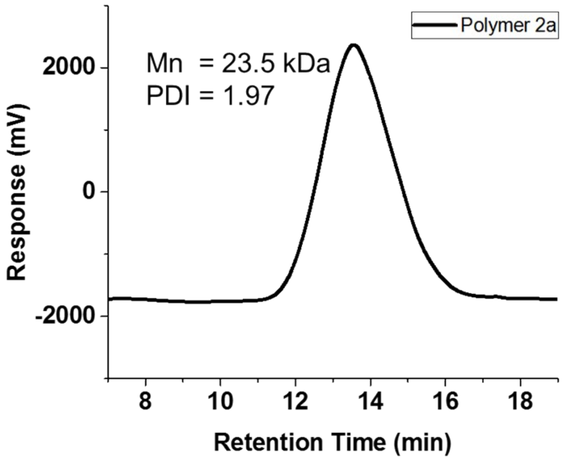

Figure S17: GPC chromatograph of polymer 2b

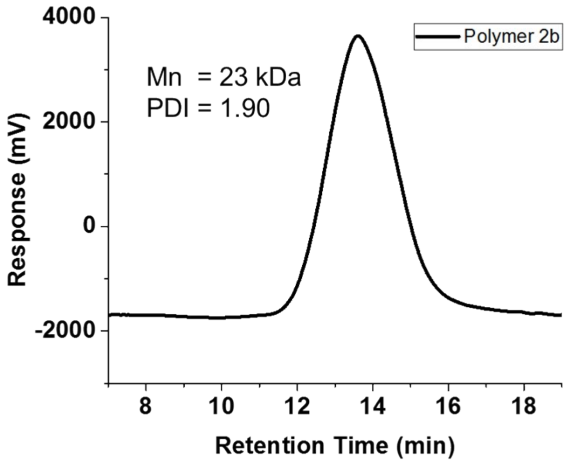

NMR Degradation Study of compound 1b

Figure S18: ^{1}H NMR of **1b** before and after heating

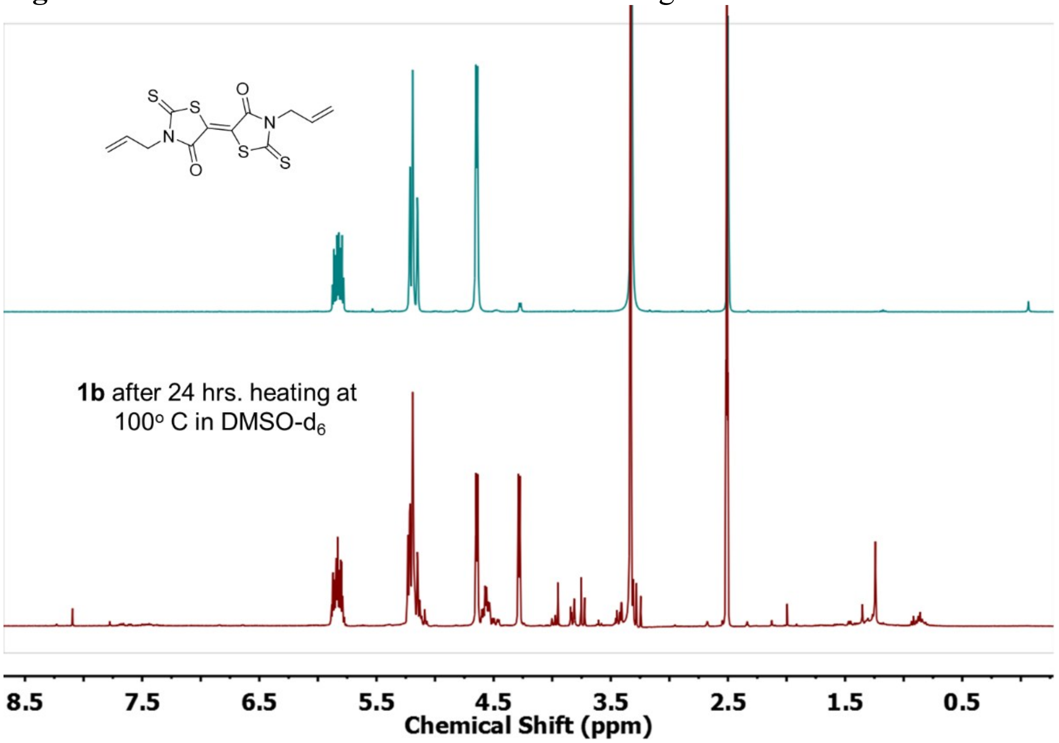

Figure S19: ^{13}C NMR of **1b** before and after heating

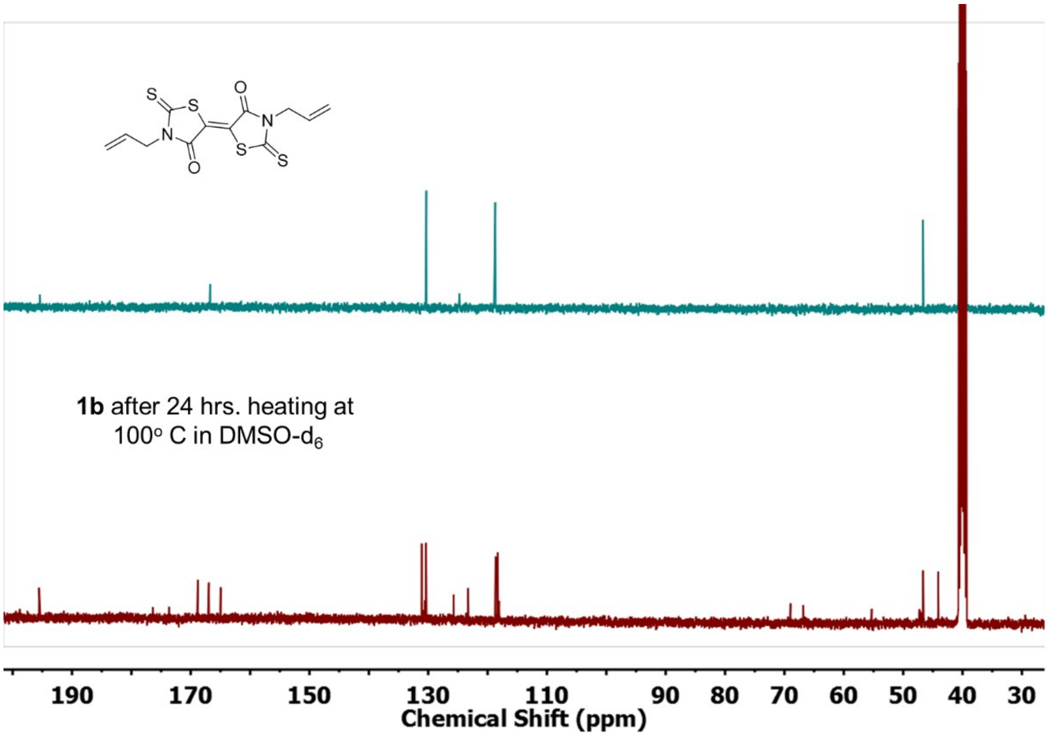

Compound **1b** was heated in DMSO-d_6 at a 0.01M concentration and heated for 24 hours at 100 ^oC. The resultant solution was concentrated and put into an NMR tube for analysis of any degradation products. From the ^1H NMR, resonances from the starting material remain while new allylic, and amide peaks begin to appear. The ^{13}C NMR shows doubling of the allyl, alkene, and methylene peaks, and new carbonyl resonances arise suggesting a break in molecular symmetry, potential from N-C or C-S bond scission.

Electrochemistry (Figure S20). Electrochemical studies were performed using a single-compartment three-electrode cell with a platinum flag as the counter electrode, a non-aqueous Ag/Ag+ reference electrode (calibrated versus ferrocene/ferrocenium (Fc/Fc^+) standard redox couple as an external standard), and a platinum button (0.02 cm^2) as the working electrode. Cyclic voltammetry measurements were carried out in 0.1 M $TBAPF_6$ DMF electrolyte solution with the analyte concentration of ca. 1-3 mg/mL. All potentials are reported *vs*. standard calomel electrode (SCE), using a 0.31 V correction factor for Fc/Fc^+ against SCE.

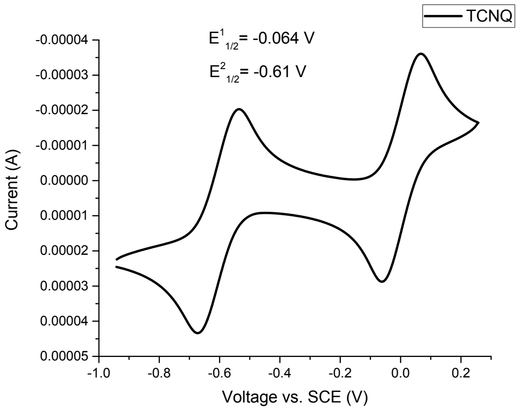
TCNQ
$E^1_{1/2}$= -0.064 V
$E^2_{1/2}$= -0.61 V
Current (A)
Voltage vs. SCE (V)

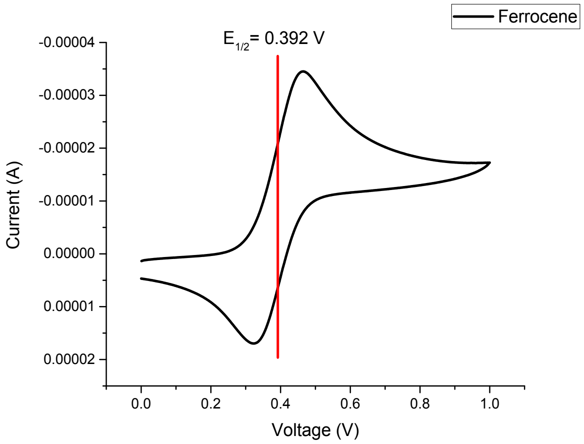
Ferrocene
$E_{1/2}$= 0.392 V
Current (A)
Voltage (V)

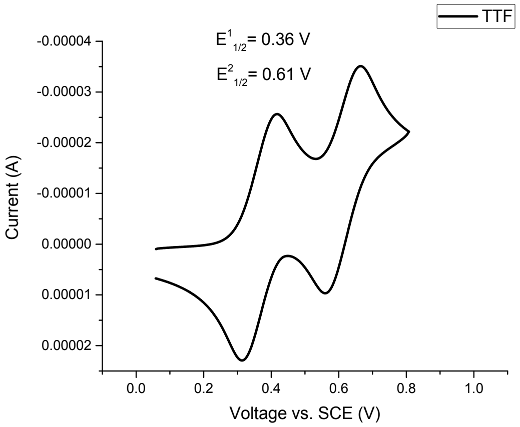
TTF
$E^1_{1/2}$= 0.36 V
$E^2_{1/2}$= 0.61 V
Current (A)
Voltage vs. SCE (V)

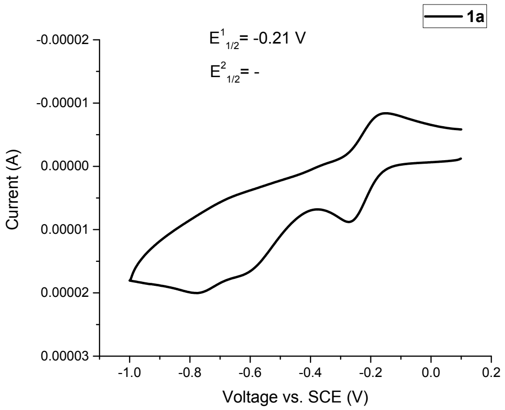
1a
$E^1_{1/2}$= -0.21 V
$E^2_{1/2}$= -
Current (A)
Voltage vs. SCE (V)

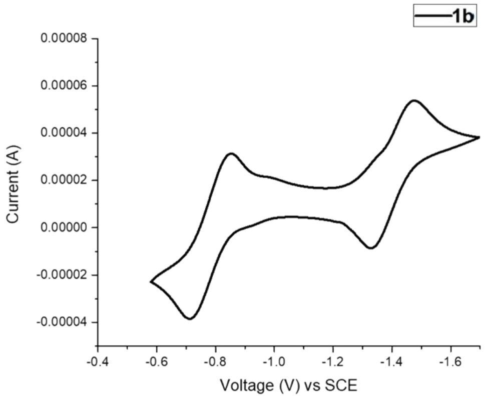
1b
Current (A)
Voltage (V) vs SCE

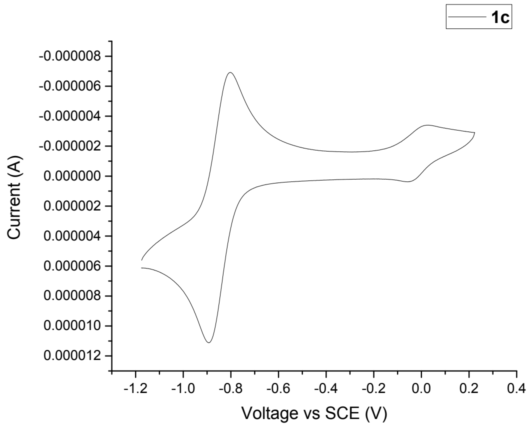
1c
Current (A)
Voltage vs SCE (V)

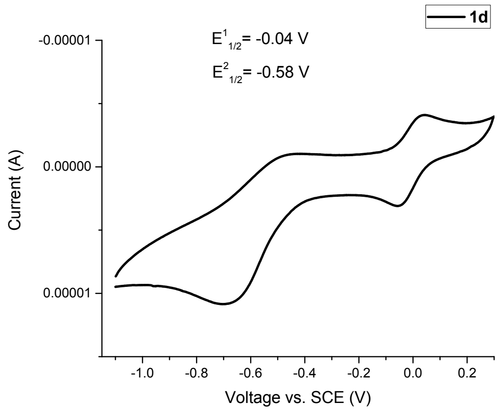

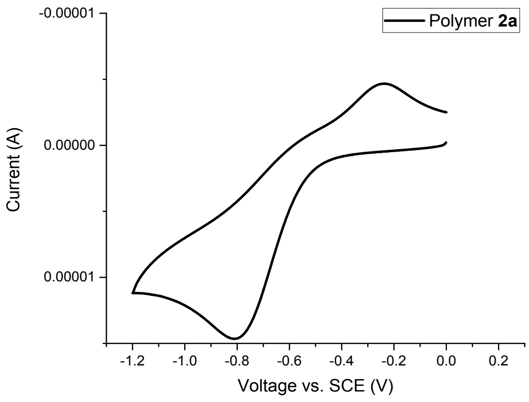

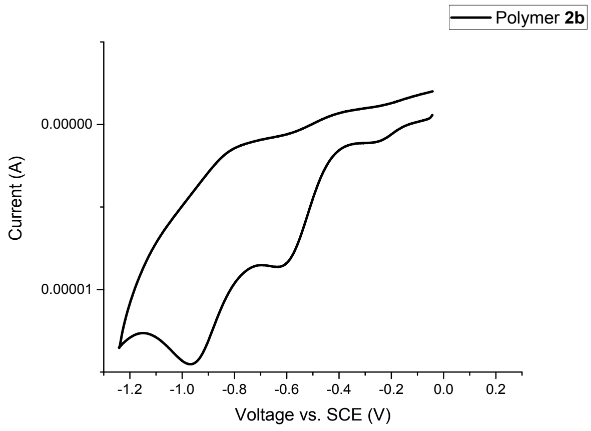

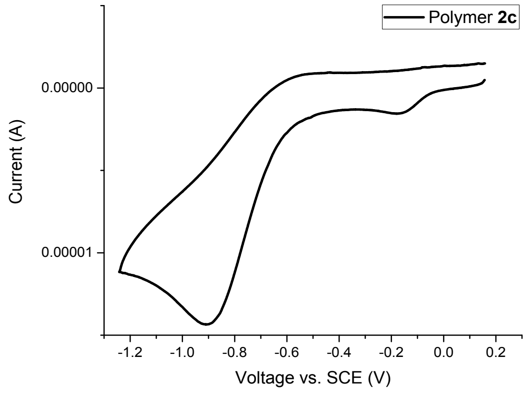

Table S1: Electrochemical reduction potentials

Compound	$E_1^{1/2}$ (V)	$E_2^{1/2}$ (V)
TTF	0.36	0.61
TCNQ	-0.064	-0.61
1a	-0.21	-
1b	-0.78	-1.4
1c	-0.04	-0.85
1d	-0.04	-0.58
2a	-0.53	-
2b	-0.5	-0.88
2c	-0.1	-0.53

Thermogravimetric Analysis (TGA) (Figure S21)

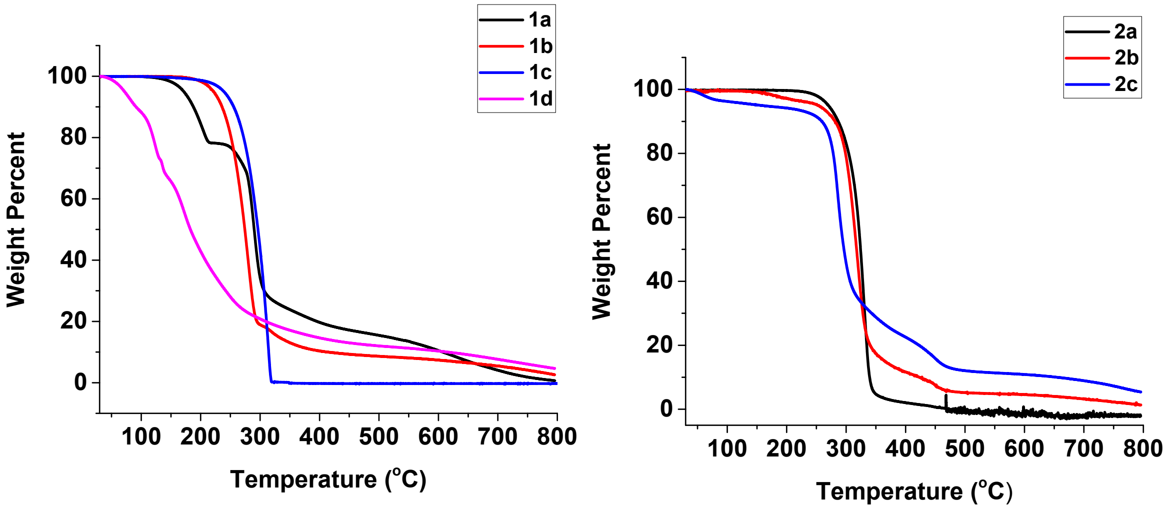

Table S2- $Td_{5\%}$ of BTs and BT-containing polymers

Compound	$T_{d5\%}$ (° C)
1a	172
1b	221
1c	239
1d	72
2a	270
2b	253
2c	158

UV-Vis Degradation Studies

Figure S22

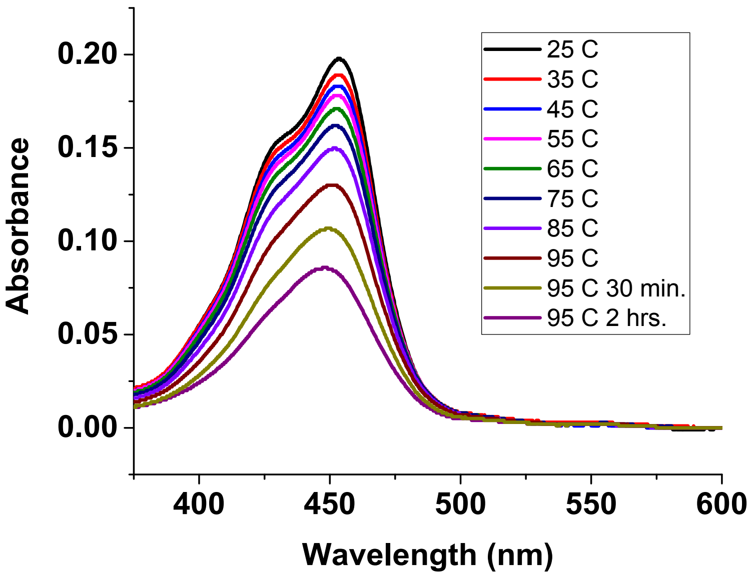

Compound **1a** heating ramp in DMF

Figure S23

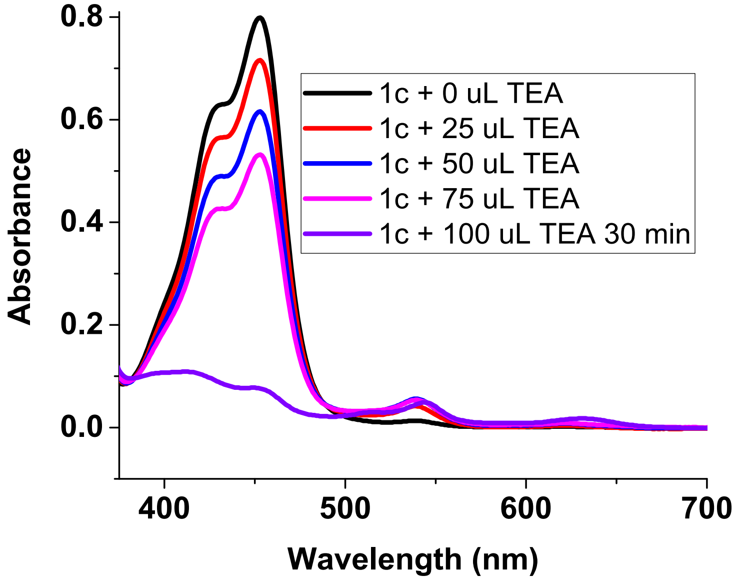

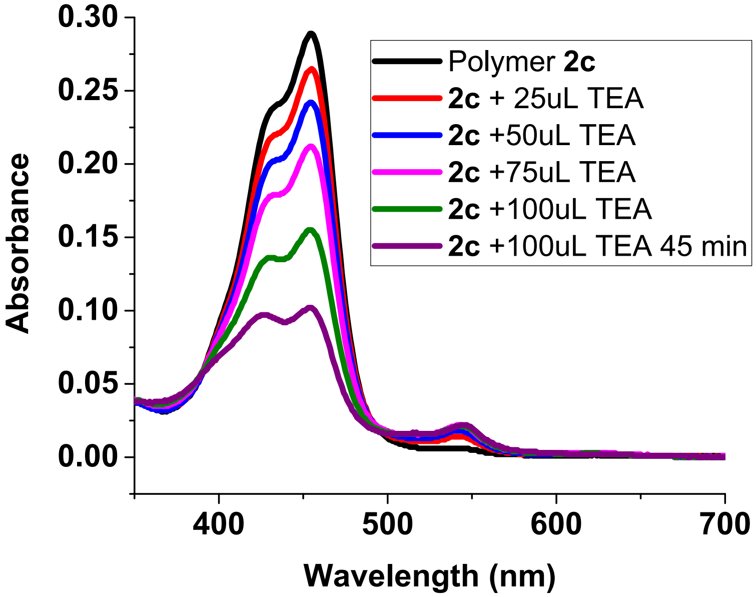

Figure S24

Figure S25 – WAXS of polymer **2b**

X-ray scattering was performed on film of polymer 2b casted from a 50 mg/mL solution onto a glass cover slip. The film was allowed to dry before being mounted onto a scattering stage for analysis. The same film was annealed on a hotplate at 100 °C for 24 hours and subsequently analyzed again for domain size changes.

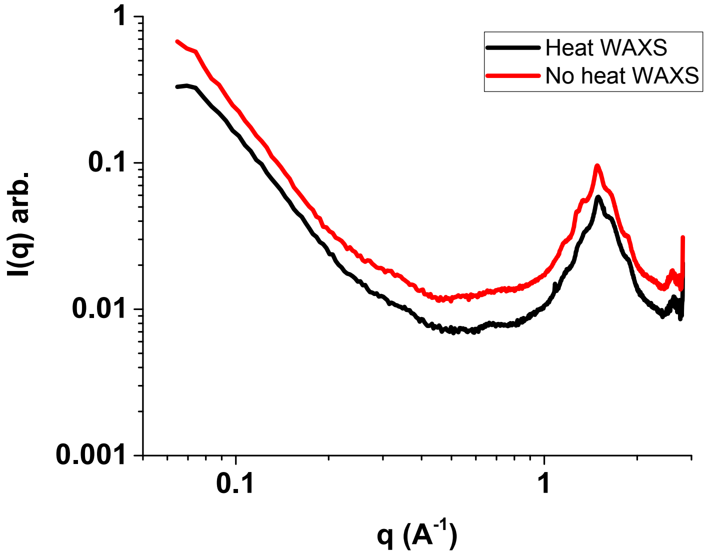

Figure S26- SAXS of polymer **2b**

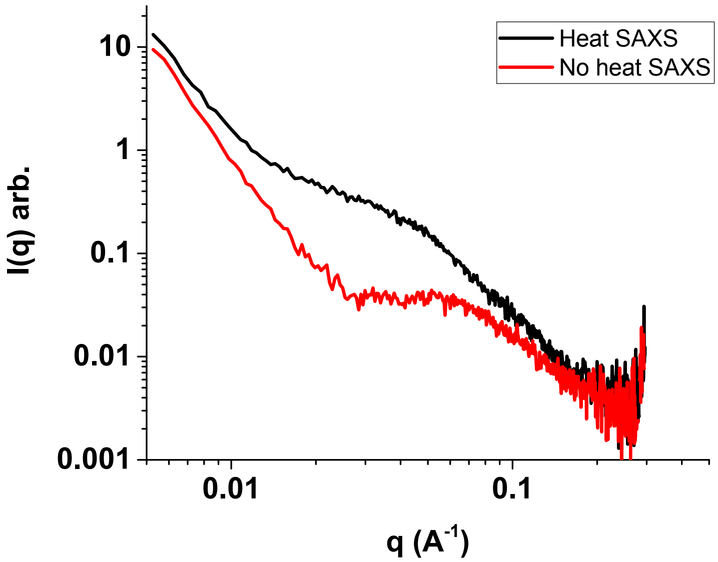

Kelvin Probe Force Microscopy

Kelvin probe force microscopy (KPFM) is a technique that measures the local surface potential by the contact potential difference between a metal-coated tip and the surface. KPFM experiments were conducted on a Digital Instruments AFM/KPFM in non-contact tapping mode, under ambient atmospheric conditions. The AFM probes were platinum-coated silicon (ANSCM-PT) used as supplied by App Nano. KPFM was performed on MoS_2 that was CVD grown on Si/SiO_2 or sapphire substrates. The substrate was scanned initially to obtain the work function of the as-grown MoS_2 and then coated with a polymer (casted from a 0.001 mg/mL solution in $CHCl_3$) and the same area was scanned again to monitor changes in the height and surface potential of the polymer-coated MoS_2. A control was carried out by scanning MoS_2 before and after drop-casting chloroform on an area of interest. The figure above shows the height and SPC before and after coating along with height and SPC histograms showing almost no change in height and SPC after addition of chloroform.

Figure S27 – Control experiment KPFM

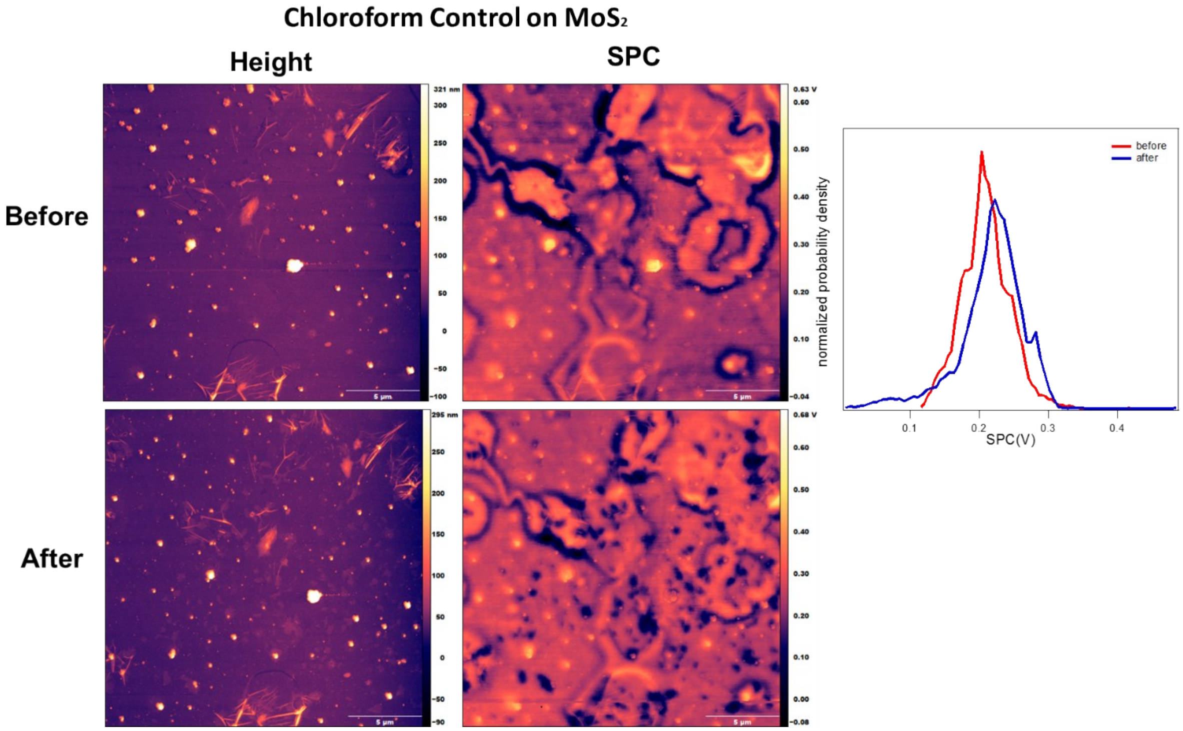

Figure S28 – Height Histograms for polymer coated MoS_2

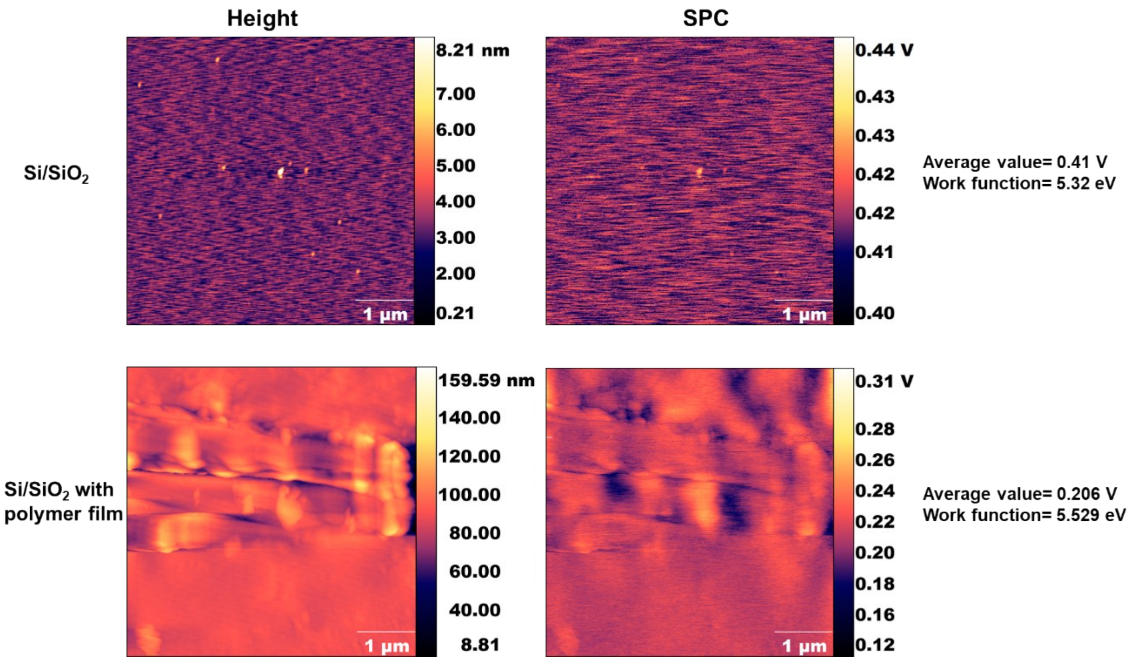

Figure S29 – Control experiment probing the work function of **2b**

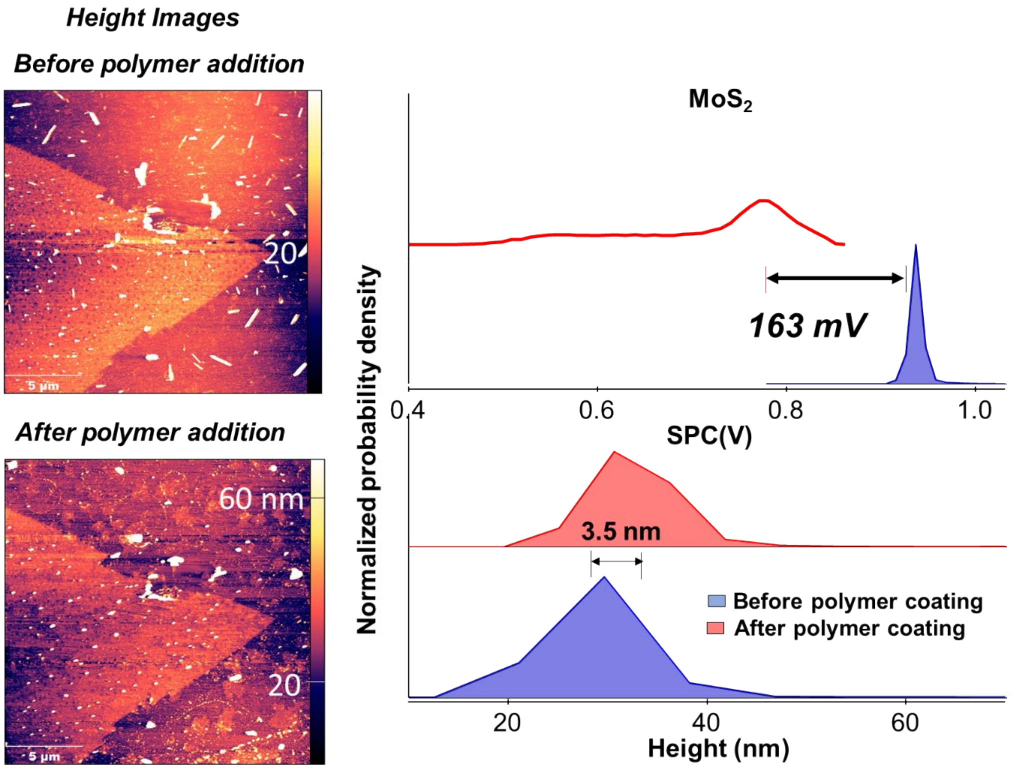

Supplement: Supplementary file 1 [file SC-009-C8SC01416G-s001.pdf]
